# Analytical and behavioral characterization of 1‐dodecanoyl‐LSD (1DD‐LSD)

**DOI:** 10.1002/dta.3691

**Published:** 2024-04-03

**Authors:** Pierce V. Kavanagh, Folker Westphal, Benedikt Pulver, Simon P. Elliott, Alexander Stratford, Adam L. Halberstadt, Simon D. Brandt

**Affiliations:** ^1^ Department of Pharmacology and Therapeutics, School of Medicine, Trinity Centre for Health Sciences St. James Hospital Dublin Ireland; ^2^ State Bureau of Criminal Investigation Schleswig‐Holstein Section Narcotics/Toxicology Kiel Germany; ^3^ Institute of Forensic Medicine, Forensic Toxicology, Medical Center, Faculty of Medicine University of Freiburg Freiburg Germany; ^4^ Elliott Forensic Consulting Birmingham UK; ^5^ Department of Analytical, Environmental and Forensic Sciences King's College London London UK; ^6^ Synex Synthetics BV Maastricht The Netherlands; ^7^ Department of Psychiatry University of California San Diego La Jolla California USA; ^8^ Research Service VA San Diego Healthcare System San Diego California USA; ^9^ School of Pharmacy and Biomolecular Sciences Liverpool John Moores University Liverpool UK

**Keywords:** head‐twitch response, LSD, new psychoactive substances, psychedelics

## Abstract

1‐Acetyl‐*N*,*N*‐diethyllysergamide (1A‐LSD, ALD‐52) was first synthesized in the 1950s and found to produce psychedelic effects similar to those of LSD. Evidence suggests that ALD‐52 serves as a prodrug in vivo and hydrolysis to LSD is likely responsible for its activity. Extension of the *N* ^1^‐alkylcarbonyl chain gives rise to novel lysergamides, which spurred further investigations into their structure–activity relationships. At the same time, ALD‐52 and numerous homologues have emerged as recreational drugs (“research chemicals”) that are available from online vendors. In the present study, 1‐dodecanoyl‐LSD (1DD‐LSD), a novel *N* ^1^‐acylated LSD derivative, was subjected to analytical characterization and was also tested in the mouse head‐twitch response (HTR) assay to assess whether it produces LSD‐like effects in vivo. When tested in C57BL/6J mice, 1DD‐LSD induced the HTR with a median effective dose (ED_50_) of 2.17 mg/kg, which was equivalent to 3.60 μmol/kg. Under similar experimental conditions, LSD has 27‐fold higher potency than 1DD‐LSD in the HTR assay. Previous work has shown that other homologues such as ALD‐52 and 1‐propanoyl‐LSD also have considerably higher potency than 1DD‐LSD in mice, which suggests that hydrolysis of the 1‐dodecanoyl moiety may be comparatively less efficient in vivo. Further investigations are warranted to determine whether the increased lipophilicity of 1DD‐LSD causes it to be sequestered in fat, thereby reducing its exposure to enzymatic hydrolysis in plasma and tissues. Further clinical studies are also required to assess its activity in humans and to test the prediction that it could potentially serve as a long‐acting prodrug for LSD.

## Graphical abstract

The development of novel lysergamides provides new opportunities for extending the understanding of their structure–activity relationships. At the same time, numerous *N* ^1^‐alkylcarbonyl derivatives of LSD emerged as recreational drugs. This study presents analytical and pharmacological data obtained for 1‐dodecanoyl‐LSD (1DD‐LSD), a novel *N* ^1^‐acylated LSD derivative. The mouse head‐twitch response data confirmed that 1DD‐LSD produces LSD‐like effects though the increased *N* ^1^‐acyl chain length led to noticeable reduction in potency (ED_50_ = 3.60 μmol/kg) compared to lower homologues.

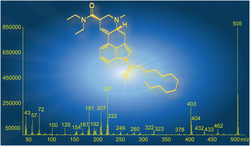

## 1 INTRODUCTION

**FIGURE 1:**
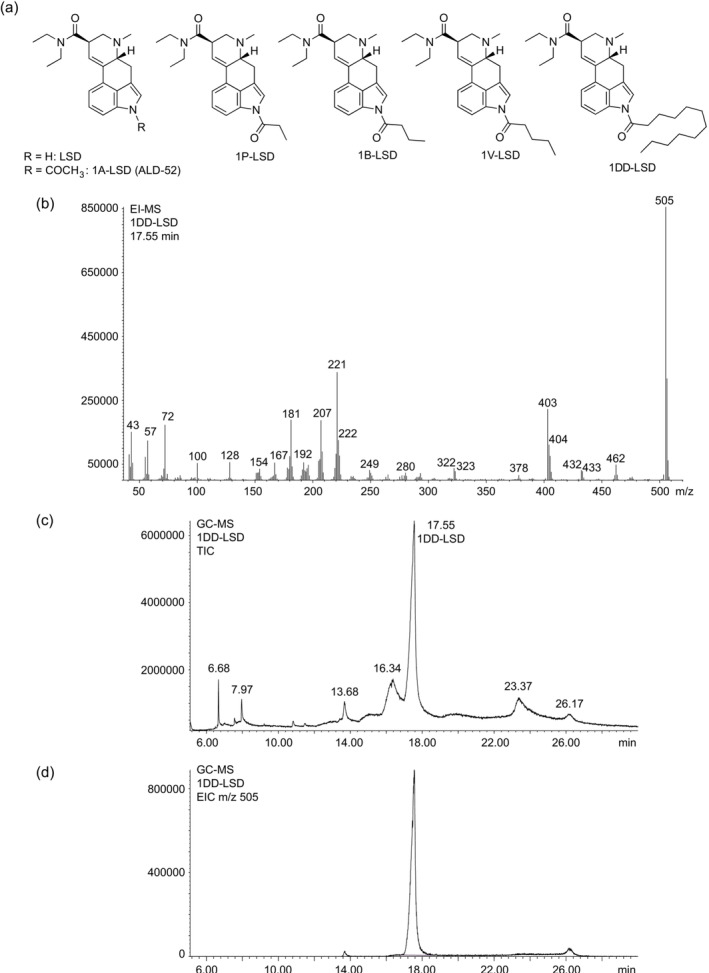
(a) Chemical structures of *N* ^1^‐acylated derivatives of lysergic acid diethylamide (LSD). (b) Electron ionization mass spectrum of 1DD‐LSD. (c) GC‐MS trace in TIC mode. (d) GC‐MS trace in EIC mode.

The synthesis of 1‐acetyl‐*N*,*N*‐diethyllysergamide (1A‐LSD, ALD‐52; Figure 1a), an *N* ^1^‐acylated derivative of lysergic acid diethylamide (LSD), was first reported by Franz Troxler and Albert Hofmann in 1957.^1^ Subsequently, ALD‐52 was distributed by Sandoz pharmaceuticals and tested in several small‐scale clinical trials, which showed it produces psychedelic effects closely mirroring those induced by LSD.^2^, ^3^, ^4^, ^5^, ^6^, ^7^ ALD‐52 is believed to serve as a prodrug in vivo, and its hydrolysis to LSD is thought to be responsible for its activity. ALD‐52 is rapidly metabolized to LSD after administration to mice.^8^ Likewise, investigations carried out in vitro showed conversion of ALD‐52 to LSD after exposure to pooled human liver S9 fractions.^9^ Although the psychedelic effects of LSD are believed to be largely mediated by activation of the 5‐HT_2A_ receptor, *N* ^1^‐acyl substitution markedly diminishes the agonist efficacy of LSD at the 5‐HT_2A_ receptor,^8^ indicating the psychoactive properties of ALD‐52 and other *N* ^1^‐acylated LSD derivatives must be largely mediated by the formation of an active metabolite.

The extent to which ALD‐52 circulated on the recreational drug market during the 1960s and the decades that followed is not clear. Claims have been made that LSD distributed under the name “orange sunshine” in the United States during the 1970s was actually ALD‐52, although this has been disputed. The first confirmed detection of ALD‐52 as a recreational drug appears to have occurred in Europe in 2016, when ALD‐52 was reported to the European Monitoring Centre for Drugs and Drug Addiction as a new psychoactive substance (NPS).^10^ Detections of ALD‐52 in Japan and Brazil were subsequently reported in the scientific literature.^11^, ^12^, ^13^

Cases of *N* ^1^‐acylated LSD derivatives containing extended linear alkylcarbonyl substituents have emerged on the “research chemical” market in recent years, and as shown in Figure 1, examples included 1P‐LSD,^12^, ^14^ 1B‐LSD,^15^, ^16^, ^17^, and 1V‐LSD, respectively.^18^, ^19^ At the same time, information on longer chain derivatives is currently unavailable, which prompted the investigation into 1‐dodecanoyl‐LSD (1DD‐LSD), an *N* ^1^‐alkylcarbonyl LSD derivative with a 12‐carbon chain (Figure 1a). Understanding the mechanism of action and structure–activity relationships of these derivatives are warranted. There is currently no indication that 1DD‐LSD is circulating on the market, but it was deemed reasonable to disseminate its analytical features.

The behavioral pharmacology of 1DD‐LSD was assessed by evaluating its effects on the head‐twitch response (HTR), a 5‐HT_2A_ receptor‐mediated head movement in mice that serves as a behavioral proxy for LSD‐like psychedelic psychopharmacology.^20^, ^21^, ^22^, ^23^ HTR testing confirmed that 1DD‐LSD is active in the paradigm but has lower potency and an extended duration of action compared to several other *N* ^1^‐acylated LSD derivatives.

## 2 EXPERIMENTAL

### 2.1 Materials

All chemicals and solvents were of analytical or HPLC grade and obtained from Aldrich (Dorset, UK). A powdered sample of 1DD‐LSD tartrate (95%) (identified as a tartrate [3:2 ratio based on ^1^H NMR]) was provided by Synex Synthetics BV, Maastricht, The Netherlands.

### 2.2 Instrumentation

#### 2.2.1 Gas chromatography–mass spectrometry (GC‐MS) (method 1)

A solution in acetonitrile (1 mg/ml, 2 μl injected) was analyzed on an Agilent 6980 GC coupled to an Agilent 5973 mass selective detector. A Rxi‐5Sil MS column (0.25 mm ID, 0.25 μm, 10 m; Restek, Derbyshire, UK) was used with helium carrier gas at a constant flow of 1 ml/min in splitless mode. The injector and transfer line were set at 250 and 280°C, respectively. The initial oven temperature program was 200°C held for 2 min and then increased at 20°C/min up to 300°C, at which it was held for 23 min. The total run time was 30 min. The mass spectrometer settings were as follows: solvent delay, 5 min; EI mode, 70 eV, range *m/z* 40–600; source temp. 230°C; quad temp. 250°C, and transfer line 280°C. Details of an alternative GC‐MS method 2 are reported as Supporting Information.

#### 2.2.2 High‐performance liquid chromatography diode array detection

A Dionex 3000 Ultimate liquid chromatography system coupled to a UV diode array detector (Thermo Fisher, St. Albans, UK) was used with a Phenomenex Synergi Fusion column (150 × 2 mm, 4 μm) protected by a 4 × 3 mm Phenomenex Synergi Fusion guard column (Phenomenex, Macclesfield, UK). The mobile phases were 70% acetonitrile with 25 mM of triethylammonium phosphate buffer (TEAP) (B) and aqueous TEAP (25 mM) buffer (A). The gradient elution commenced with 4% B and ramped to 70% B over 15 min and then held for 3 min, resulting in a total acquisition time of 18 min at a flow rate of 0.6 ml/min. The diode array detection window was set at 200–595 nm (collection rate of 2 Hz).

#### 2.2.3 Ultra‐high‐performance liquid chromatography–electrospray ionization tandem mass spectrometry (UHPLC‐QTOF‐MS/MS)

UHPLC‐QTOF‐MS/MS data were obtained from an Agilent 6540 UHD Accurate‐Mass QTOF LC‐MS system coupled to an Agilent 1290 Infinity UHPLC system (Agilent, Cheshire, UK). Separation was achieved using an Agilent Zorbax Eclipse Plus C18 column (100 × 2.1 mm, 1.8 μm) (Agilent, Cheadle, UK). Mobile phases consisted of acetonitrile (containing 1% formic acid) and 1% formic acid in water. The column temperature was set at 40°C and data were acquired for 5.5 min. The flow rate was (0.6 ml/min). The gradient was set at 5–70% acetonitrile over 3.5 min, then increased to 95% acetonitrile in 1 min and held for 0.5 min before returning to 5% acetonitrile in 0.5 min. QTOF‐MS data were acquired in positive ion mode scanning from m/z 100–1000 with and without auto MS/MS fragmentation. Ionization was achieved with an Agilent JetStream electrospray source and infused internal reference masses. Ion source parameters were gas temperature 325°C, drying gas 10 L/min, and sheath gas temperature 400°C. Internal reference ions at m/z 121.05087 and m/z 922.00979 were used for calibration purposes. The sample was dissolved in methanol at a concentration of 10 μg/ml.

#### 2.2.4 Nuclear magnetic resonance spectroscopy (NMR)

NMR spectra (^1^H at 600 MHz; ^13^C at 150 MHz) of the powdered sample (10 mg, 0.75 ml solvent) were recorded using a Bruker AVANCE III 600 MHz spectrometer (Bruker UK Ltd, Coventry, UK) in DMSO‐*d*_6_. Experiments were carried out at 298 K with a 5 mm PA BBO probe with z‐gradient. Spectra were referenced to residual solvent, and assignments were supported by both 1D and 2D experiments.

### 2.3 Animal pharmacology

Male C57BL/6J mice (6–8 weeks old) were obtained from Jackson Laboratories (Bar Harbor, ME, USA) and housed up to four per cage with a reversed light cycle (lights on at 1900 h, off at 0700 h). Food and water were provided ad libitum, except during behavioral testing. Testing was conducted between 1000 and 1830 h. All animal experiments were carried out in accordance with NIH guidelines and were approved by the UCSD animal care committee. The HTR was assessed using a head‐mounted magnet and a magnetometer detection coil.^23^ Mice were anesthetized, a small incision was made in the scalp, and a small neodymium magnet was attached to the dorsal surface of the cranium using dental cement. Following a 2‐week recovery period, HTR experiments were carried out in a well‐lit room with at least 7 days between sessions to avoid carryover effects. Mice were injected IP (5 ml/kg injection volume) with vehicle (water containing 16% dimethylsulfoxide) or 1DD‐LSD (1, 2, 4, or 8 mg/kg), and then activity was recorded in a glass cylinder surrounded by a magnetometer coil for 60 min. Coil voltage was low‐pass filtered (2‐kHz cutoff frequency), amplified, digitized (20‐kHz sampling rate, 16‐bit ADC resolution), and saved to disk using a PowerLab 8/35 data acquisition system with LabChart software ver. 8.1.16 (ADInstruments, Colorado Springs, CO, USA). To detect head twitches, events in the recordings were transformed to scalograms, deep features were extracted using the deep convolutional neural network ResNet‐50, and then the images were classified using a support vector machine (SVM).^24^ Total head‐twitch counts were analyzed using a one‐way ANOVA. HTR counts were also binned in 2‐min blocks and analyzed using a two‐way ANOVA (drug × time). *Post hoc* comparisons were made using Dunnett's test. Significance was demonstrated by surpassing an α‐level of 0.05. ED_50_ values and 95% confidence intervals were calculated using nonlinear regression.

## 3 RESULTS AND DISCUSSION

### 3.1 Analytical features

The electron ionization (EI) mass spectrum of 1DD‐LSD is shown in Figure 1b, and proposed fragmentation pathways are included as Supporting Information adapted from those suggested previously for 1P‐LSD,^14^ 1B‐LSD,^15^ and 1V‐LSD, respectively.^18^ Key ions specifically related to 1DD‐LSD (attachment of the *N* ^1^‐dodecanoyl group) included the retro‐Diels–Alder fragment at *m/z* 462, characteristically formed by a neutral loss of 43 u (*N*‐methylmethanimine) that reflected the presence of the *N* ^6^‐methyl group. One of the fragment clusters frequently seen with LSD‐type compounds (but also certain isomers) include those detected at *m/z* 218–224 with one example possibly being *m/z* 222, formed after a neutral loss of *N*,*N*‐diethylformamide.^25^ In the case of 1DD‐LSD, the mass shift related to the presence of the *N* ^1^‐dodecanoyl group (182 u) was detected at *m/z* 404, which was part of the mass‐shifted cluster at *m/z* 403–406. The loss of the *N*,*N*‐diethylamino radical (C_4_H_10_N^•^, 72 u) might have produced the oxonium species at *m/z* 433, which also indicated that it might have served as an originator of *m/z* 250 following the loss of the *N* ^1^‐acyl group. Low‐mass ions at *m/z* 57 and 43 were also detected, which suggested potential secondary fragmentations from fragmentations of the *N* ^1^‐acyl group (Figure 1b). The EI mass spectrum of 1V‐LSD also showed an *m/z* 57 species, although it was hypothesized to have formed from a neutral loss of CO from the oxonium ion at *m/z* 85.^18^

**FIGURE 2:**
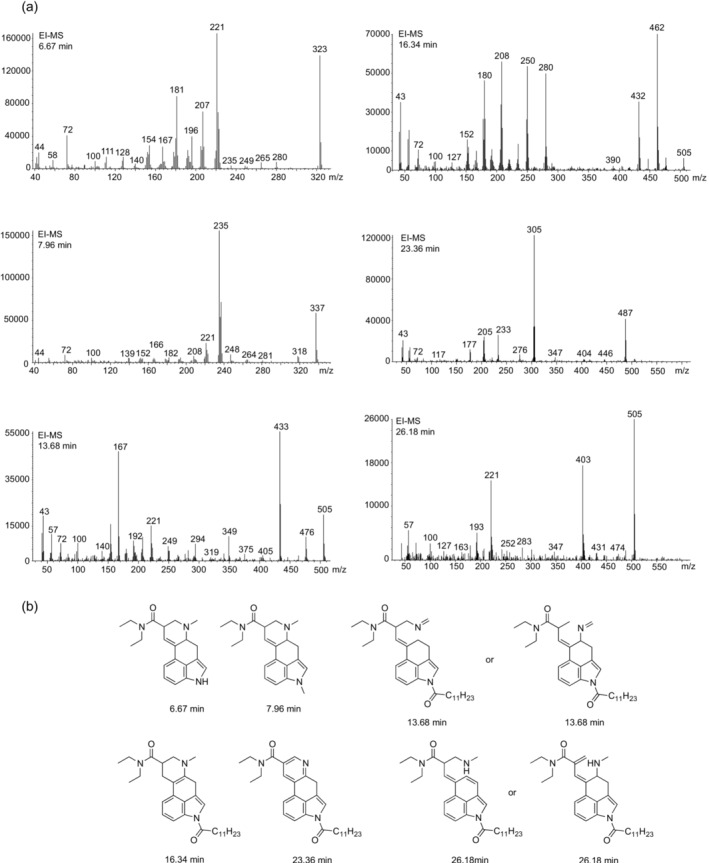
(a) Electron ionization mass spectra recorded from GC‐MS peaks shown in Figure 1c. (b) Tentative structures of potentially GC‐induced artifacts based on mass spectral considerations (Supporting Information).

Results from GC‐MS analysis of 1DD‐LSD are shown in Figure 1c (TIC) and 1d (EIC), respectively. In addition to 1DD‐LSD, six additional peaks were detected in full scan mode. The EI mass spectra and proposed identities are summarized in Figure 2, though it should be noted that these assignments remain hypothetical (with the exception of LSD) because reference material was unavailable. Attempts to rationalize their proposed identification involved suggested fragmentation pathways (Supporting Information) adapted from work carried out previously with 1V‐LSD.^18^ The extent to which these detected peaks (Figure 1c) reflect synthesis‐related impurities is uncertain, but it was also deemed reasonable to consider their artificial formation during GC‐based analysis because they were undetectable under LC‐MS conditions (with the exception of LSD). As shown in the Supporting Information, 1DD‐LSD was also analyzed using an alternative GC‐MS method 2 where an increase of the GC oven temperature to 340°C was evaluated. The reason why the GC method 1 included here resulted in a comparatively short retention time for 1DD‐LSD is that the GC column (originally 30 m long) was shortened to 10 m, which was not the case for GC‐MS method 2. Previous reports suggest that use of solvents such as methanol or ethanol has a noticeable impact on the formation of LSD when ALD‐52 and 1P‐LSD were subjected to GC‐MS analysis.^26^ Other contributing factors impacting on the detection the compounds reported here (Figure 2) may have included varying conditions of the GC liner (including active sites) and injection port temperatures. However, the detection of LSD by GC‐MS was also corroborated by the analysis by LC‐MS (Supporting Information).

**FIGURE 3:**
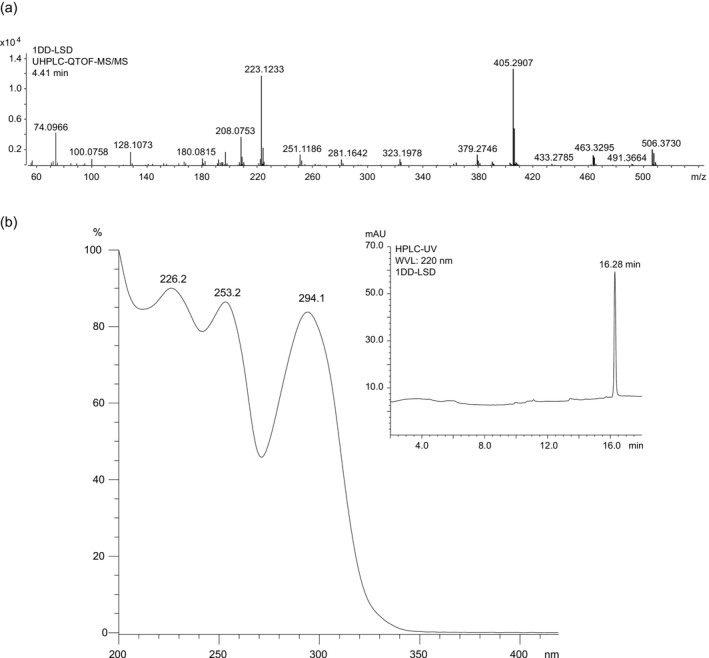
(a) Electrospray ionization QTOF tandem mass spectrum of 1DD‐LSD. (b) Ultraviolet spectrum of 1DD‐LSD recorded from the chromatographic peak at 16.28 min (insert).

Electrospray ionization QTOF tandem mass spectral data for 1DD‐LSD are shown in Figure 3a together with proposed formations of product ions (included as Supporting Information), which were based on data reported previously 1P‐LSD,^14^ 1B‐LSD,^15^ and 1V‐LSD, respectively.^18^ The majority of the ions detected were as expected and compared well with those reported for many other lysergamides abundantly reported elsewhere. However, key ions reflecting the presence of the *N* ^1^‐dodecanoyl substituent included the retro‐Diels–Alder fragment at *m/z* 463.3295 (C_30_H_43_N_2_O_2_^+^, Δm: −5.18 ppm), *m/z* 405.2907 (C_27_H_37_N_2_O^+^, Δm: 1.73 ppm, possibly formed after loss of CO from an oxonium ion), and *m/z* 379.2746 (C_25_H_35_N_2_O^+^, Δm: 0.53 ppm) (Supporting Information). The retention time of 1DD‐LSD under HPLC‐UV conditions was 16.28 min, and the ultraviolet spectrum recorded with the diode array detector (Figure 3b) was reminiscent of other *N* ^1^‐acylated lysergamides with three peak maxima at 226, 253, and 294 nm.^11^, ^12^, ^14^, ^15^, ^16^, ^18^, ^19^, ^26^, ^27^

Table 1 provides a summary of the ^1^H and ^13^C NMR data recorded for 1DD‐LSD, with full spectra included as Supporting Information. Assignments are supported by 2D NMR data and are consistent with the structure of 1DD‐LSD. The majority of the protons associated with the 12‐carbon chain could not be fully resolved, although the carbons could be identified individually. Between the H‐9 integral and the tartaric acid singlet at 4.23 ppm, a 1:1 ratio would normally be expected to reflect a 2:1 ratio (lysergamide: tartaric acid). However, since the integral showed 1.36 protons instead of 1.0 (presumably due to excess tartaric acid), the 1DD‐LSD: tartrate molar ratio was estimated to be ~3:2 instead of 2:1.

**TABLE 1:** ^1^H and ^13^C NMR data for 1DD‐LSD tartrate (3:2) in DMSO‐*d*_6_ at 600/150 MHz.

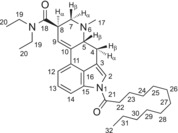		
No.	^13^C [δ/ppm]	^1^H [δ/ppm]
1	—	—
2	120.05	7.60 (d, *J* = 1.8 Hz, 1 H)
3	116.05	—
4	26.14	2.47–2.41 (m, H‐4α, 1 H) *Partially overlapping with H‐17 3.48 (dd, *J* = 15.4, 5.5 Hz, H‐4β, 2 H) *Partially overlapping with H‐19
5	61.84	3.07–3.02 (m, H‐5β, 1 H)
6	—	—
7	55.35	3.00 (dd, *J* = 11.0, 4.8 Hz, H‐7α, 1 H) 2.60 (t, *J* = 10.8 Hz, H‐7β, 1 H)
8	38.92	3.85–3.78 (m, H‐8α, 1 H)
9	121.83	6.34 (s, 1 H)
10	133.50	—
11	127.80	—
12	116.57	7.34 (d, *J* = 7.4 Hz, 1H)
13	125.91	7.30 (t, *J* = 7.7 Hz, 1H)
14	114.83	8.00 (d, *J* = 7.8 Hz, 1H)
15	133.15	—
16	127.56	—
17	43.14	2.48 (s, 3 H) *Overlapping with solvent and partially overlapping with H‐4α
18	170.39	—
19	41.57	3.44 (q, *J* = 7.1 Hz, 2 H)
19	39.48	3.31 (AB qq, *J* = 13.9, 7.0 Hz, 2 H) *Coalescing with broad water signal
20	14.83	1.18 (t, *J* = 7.1 Hz, 3 H)
20	13.06	1.06 (t, *J* = 7.1 Hz, 3 H)
21	171.84	—
22	34.70	2.96 (t, *J* = 7.3 Hz, 2 H)
23	24.17	1.67 (p, *J* = 7.4 Hz, 2 H)
24	31.28, 28.99, 28.98, 28.92, 28.79, 28.70, 28.48, 22.09	1.39–1.34 (m, 2 H)
25–31		1.20–1.34 (m, 14 H)
32	13.95	0.85 (t, *J* = 7.0 Hz, 3 H)
TA	173.27	—
TA	71.99	4.23 (s, ~1.4 H)

Abbreviation: TA, tartaric acid.

### 3.2 HTR

**FIGURE 4:**
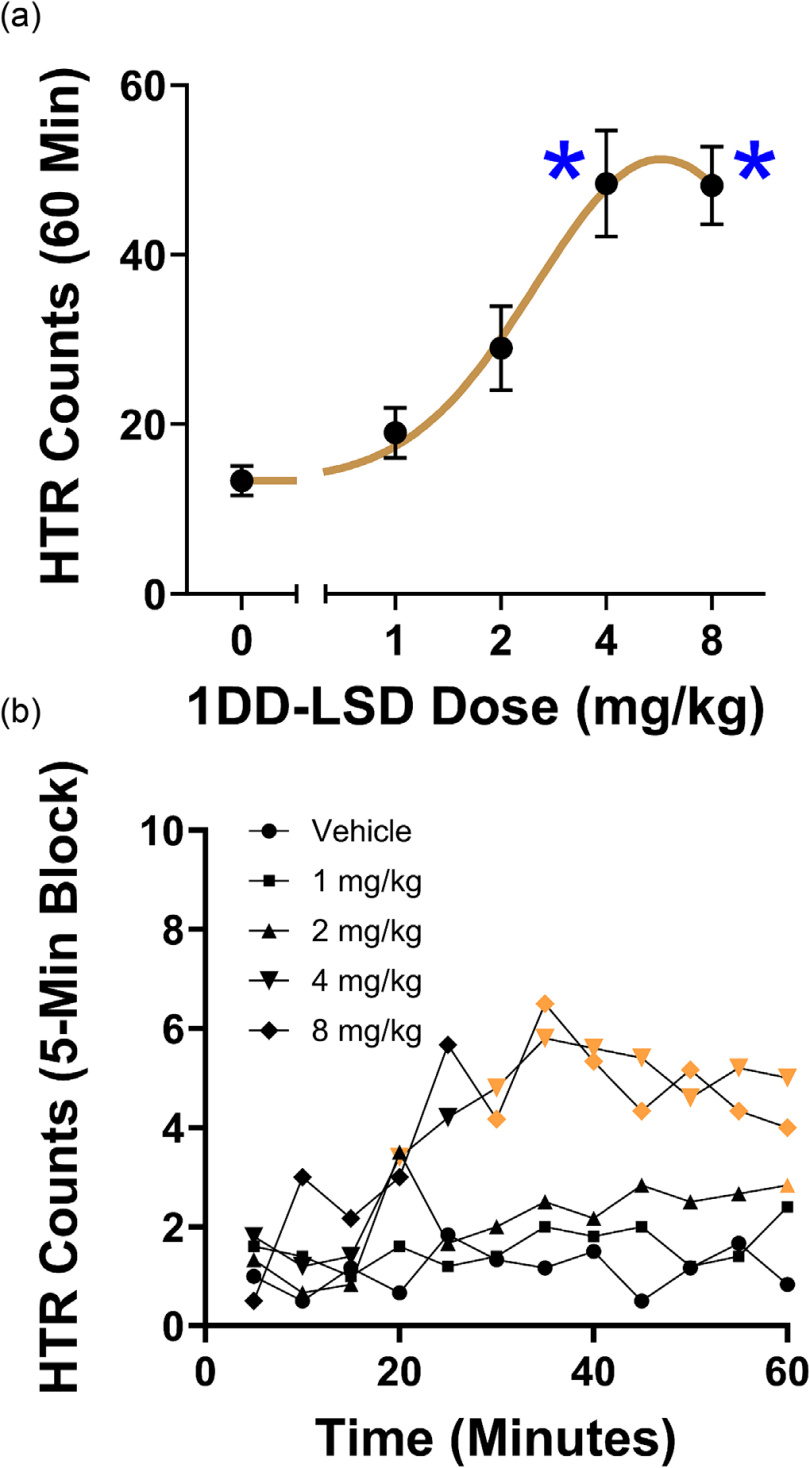
(a) Effect of 1‐dodecanoyl‐LSD (1DD‐LSD) on the head twitch response. Data are presented as group means ± SEM for the entire 60‐min test session. *p < 0.0001, significant difference from vehicle control group (Tukey's test). (b) Time course of the head twitch response induced by 1DD‐LSD. Data are presented as group means during 5‐min time blocks. The time blocks where there were significant differences from the vehicle control group are identified using colored symbols, *p* < 0.05 (Dunnett's test).

1DD‐LSD was tested in the mouse HTR assay to determine whether it produces LSD‐like effects in vivo. Previous studies have shown that the HTR in male C57BL6J mice is highly predictive of psychedelic potential in humans.^20^ Administration of 1DD‐LSD induced the HTR (*F*_4,22_ = 14.90, *p* < 0.0001), with the 4 and 8 mg/kg doses producing a significant increase in HTR counts over baseline levels (Figure 4a). The median effective dose (ED_50_) for 1DD‐LSD was 2.17 (95% CI 1.61–2.93) mg/kg, which is equivalent to 3.60 μmol/kg. When tested under similar experimental conditions, LSD induced the HTR with an ED_50_ of 132.8 nmol/kg,^23^ which is 27‐fold higher than the potency of 1DD‐LSD.

Similar to 1DD‐LSD, several other *N* ^1^‐alkylcarbonyl‐substituted LSD derivatives including ALD‐52, 1P‐LSD, 1B‐LSD, and 1V‐LSD induced the HTR in mice. Although ALD‐52 and 1P‐LSD exhibited weak agonist activity at the 5‐HT_2A_ receptor, they are hydrolyzed to LSD under in vitro and in vivo conditions and probably act as prodrugs.^8^, ^28^ Given its activity in HTR, it is reasonable to assume that 1DD‐LSD probably also serves as an LSD prodrug. However, ALD‐52 (ED_50_ = 297.2 nmol/kg^8^), 1P‐LSD (ED_50_ = 349.6 nmol/kg^14^), 1B‐LSD (ED_50_ = 976.7 nmol/kg^15^), and 1V‐LSD (ED_50_ = 373 nmol/kg^18^) have considerably higher potency than 1DD‐LSD in mice, which may indicate that the hydrolysis of 1DD‐LSD is relatively inefficient. 1DD‐LSD is also a much more lipophilic molecule due to the presence of a 1‐dodecanoyl group, which may cause it to be sequestered in fat, thereby reducing its exposure to esterases in plasma and tissues.

It was previously reported that *N* ^1^‐alkylcarbonyl‐substitution generally had relatively little effect on the time course of the HTR induced by LSD. The response to LSD^23^ and 1B‐LSD^15^ peaked approximately 5–10 min after IP injection. The max response induced by 1P‐LSD was slightly delayed in comparison (~10–20 min after IP administration^14^). To assess the time course of 1DD‐LSD, the HTR data were binned and analyzed in 5‐min blocks. There was a main effect of drug (*F*_4,23_ = 13.70, *p* < 0.0001) and an interaction between drug and time (*F*_44,253_ = 2.501, *p* < 0.0001). The response to 1DD‐LSD (Figure 4b) peaked 35 min post‐injection, and the 4 mg/kg and 8 mg/kg doses were active throughout the second half of the test session (i.e., 30–60 min after injection) based on post hoc comparisons. If 1DD‐LSD is stored in fat and hydrolyzed over an extended period, then it may be possible to use 1DD‐LSD as a depot form of LSD.^29^

## 4 CONCLUSION

The analytical and pharmacological data obtained for 1DD‐LSD may be of interest to scientists and clinicians engaged in research focusing on psychedelic drugs and other recreational substances. The HTR data confirmed that 1DD‐LSD produces behavioral effects mirroring those induced by serotonergic psychedelics such as LSD. However, the increased *N* ^1^‐acyl chain length in 1DD‐LSD led to noticeable reduction in potency compared to lower homologues, such as ALD‐52, 1P‐LSD, 1B‐LSD, and 1V‐LSD. Further clinical testing is necessary to assess the abuse potential of 1DD‐LSD and to evaluate its pharmacological interactions and the qualitative nature of its effects in animals and humans. In addition, the pharmacokinetic properties of 1DD‐LSD need to be investigated. Based on the HTR data, 1DD‐LSD appears to have an extended duration of action compared to LSD and other *N* ^1^‐acyl‐substituted homologs, possibly because it is sequestered in fat, potentially protecting it from enzymatic hydrolysis. Further studies are warranted to investigate whether depot forms of 1DD‐LSD should be developed by adjusting the formulation or by creating lipophilic esters that are sequestered in fat and slowly released and metabolized.

## Supporting information


**Data S1.** Supporting Information.
