# Supplementary material for: Analytical and behavioral characterization of 1‐dodecanoyl‐LSD (1DD‐LSD)
Source: Drug Test Anal. 2024 Apr 3;17(1):101–9. doi: 10.1002/dta.3691 (PMC11730435; doi:10.1002/dta.3691)

Analytical and behavioral characterization of 1-dodecanoyl-LSD (1DD-LSD)

Pierce V. Kavanagh,[1] Folker Westphal,[2] Benedikt Pulver,[2,3] Simon P. Elliott,[4,5] Alexander Stratford,[6] Adam L. Halberstadt,[7,8] Simon D. Brandt,[9*]

[1] *Department of Pharmacology and Therapeutics, School of Medicine, Trinity Centre for Health Sciences, St. James Hospital, Dublin 8, Ireland*

[2] *State Bureau of Criminal Investigation Schleswig-Holstein, Section Narcotics/Toxicology, Kiel, Germany*

[3] *Institute of Forensic Medicine, Forensic Toxicology, Medical Center, Faculty of Medicine, University of Freiburg, Freiburg, Germany.*

[4] *Elliott Forensic Consulting, Birmingham, UK*

[5] *Department of Analytical, Environmental and Forensic Sciences, King's College London, London SE1 9NH, UK*

[6] *Synex Synthetics BV, Maastricht, The Netherlands*

[7] *Department of Psychiatry, University of California San Diego, La Jolla, USA*

[8] *Research Service, VA San Diego Healthcare System, San Diego, USA*

[9] *School of Pharmacy and Biomolecular Sciences, Liverpool John Moores University, Byrom Street, Liverpool, L3 3AF, UK*

* Correspondence to: Simon D. Brandt, School of Pharmacy and Biomolecular Sciences, Liverpool John Moores University, Byrom Street, Liverpool, L3 3AF, UK. E-Mail: s.brandt@ljmu.ac.uk

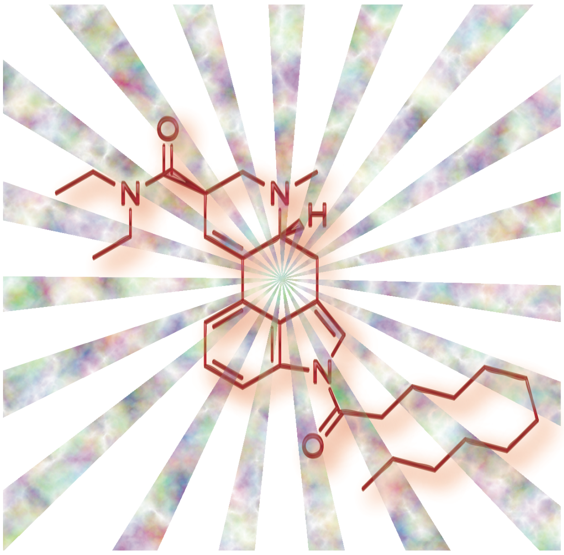

**Content**	**Page**
Proposed EI-MS fragmentation pathways (1DD-LSD)	S3–S6
Tentative identification of GC-induced artifacts (GC-MS method 1)	S7–S11
Gas chromatography-mass spectrometry (GC-MS) – method 2	S12
GC-MS data (method 2)	S13
Proposed ESI-QTOF-MS/MS fragmentation pathways for 1DD-LSD	S14
LC-ESI-linear ion trap (IT) tandem mass spectrum of 1DD-LSD	S15
LC-ESI-IT-MS chromatographic trace of 1DD-LSDand detection of LSD	S16
Attenuated total reflection-infrared spectroscopy (ATR-IR) method and spectrum	S17
^{1}H NMR	S18–S21
^{1}H/^{1}H COSY	S22–S25
^{13}C DEPTQ	S26–S27
^{1}H/^{13}C HSQC	S28–S30
^{1}H/^{13}C HMBC	S31–S34

Proposed EI-MS fragmentation pathways for 1DD-LSD

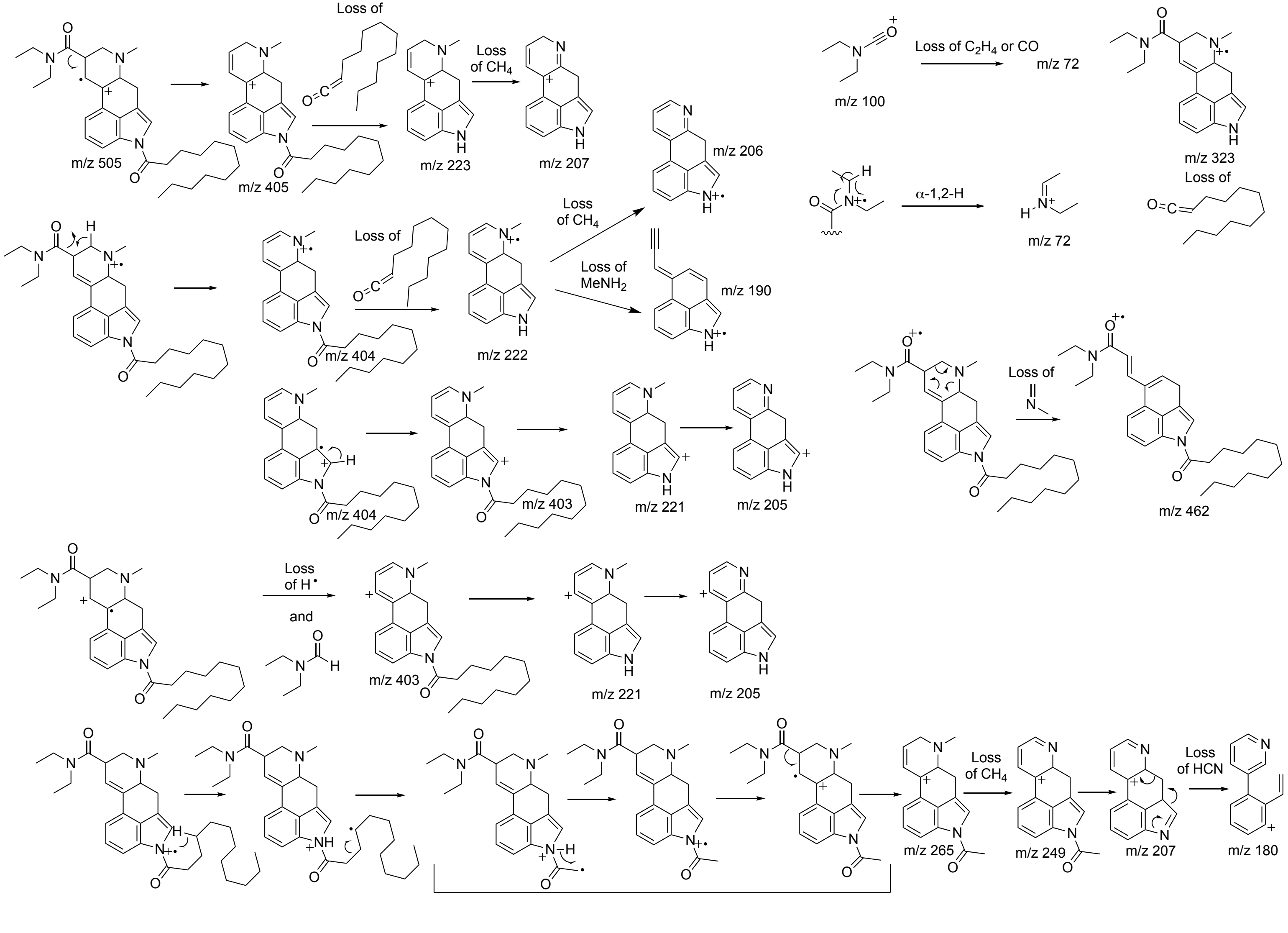

Proposed EI-MS fragmentation pathways for 1DD-LSD

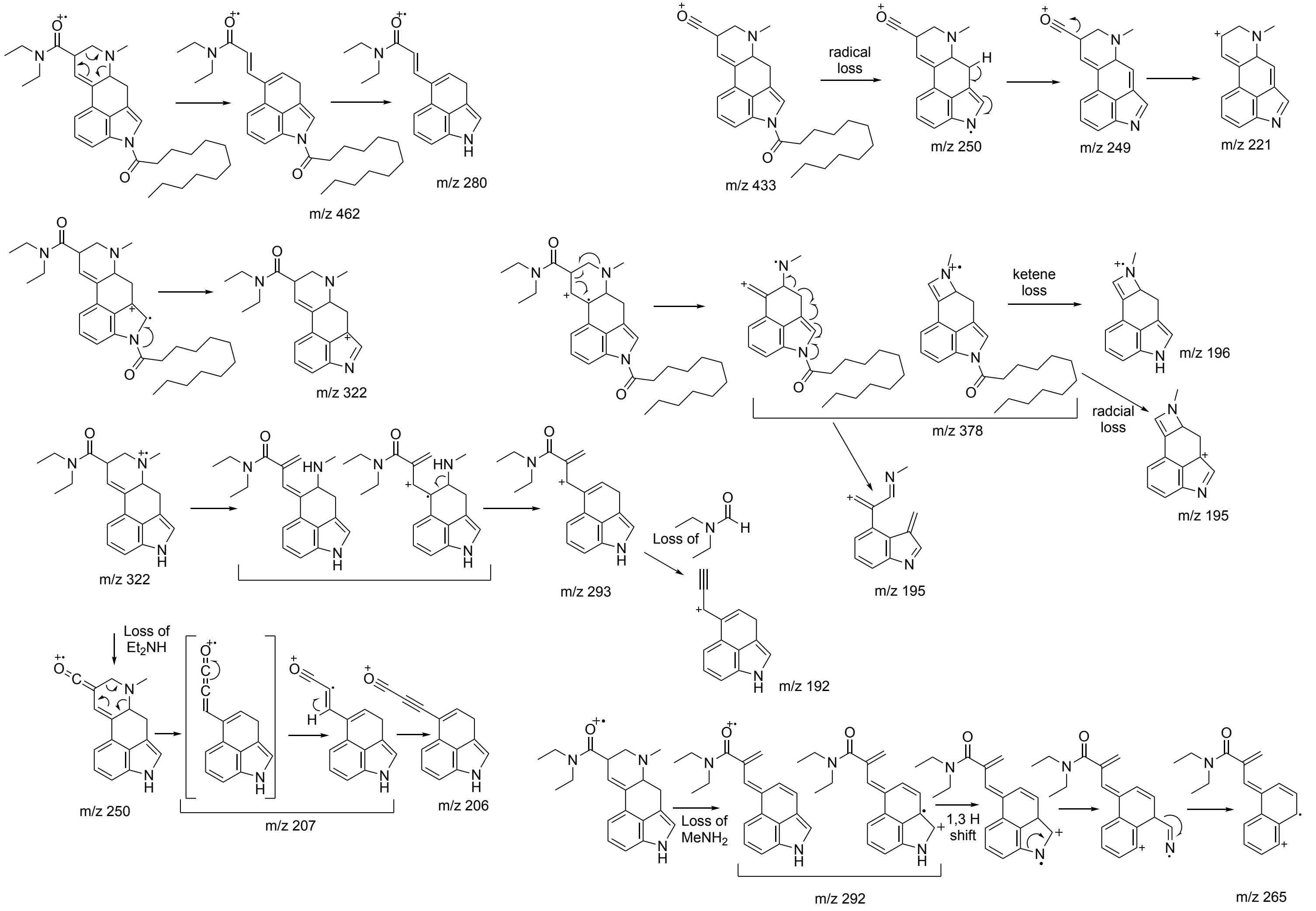

Proposed EI-MS fragmentation pathways for 1DD-LSD

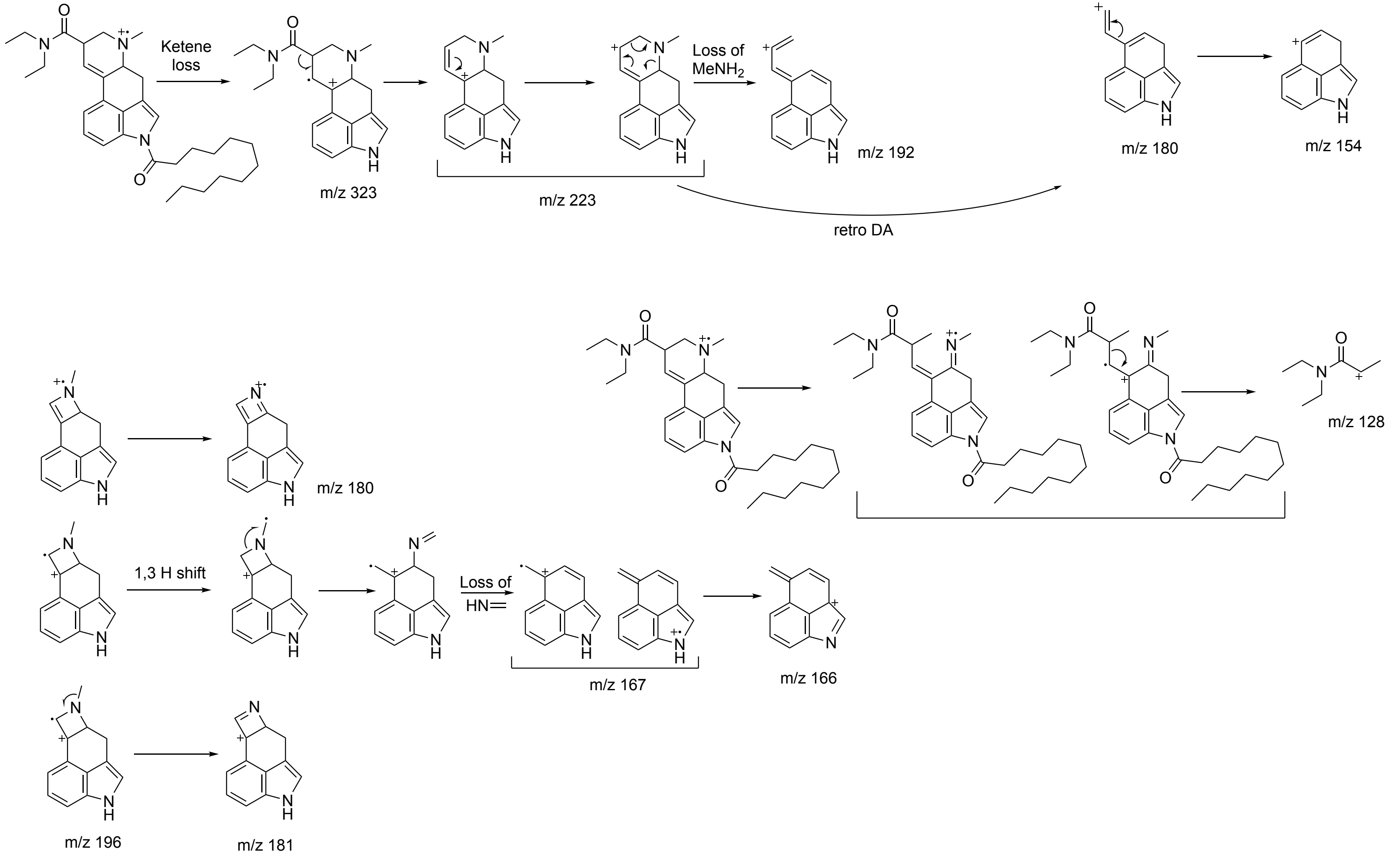

Proposed EI-MS fragmentation pathways for 1DD-LSD

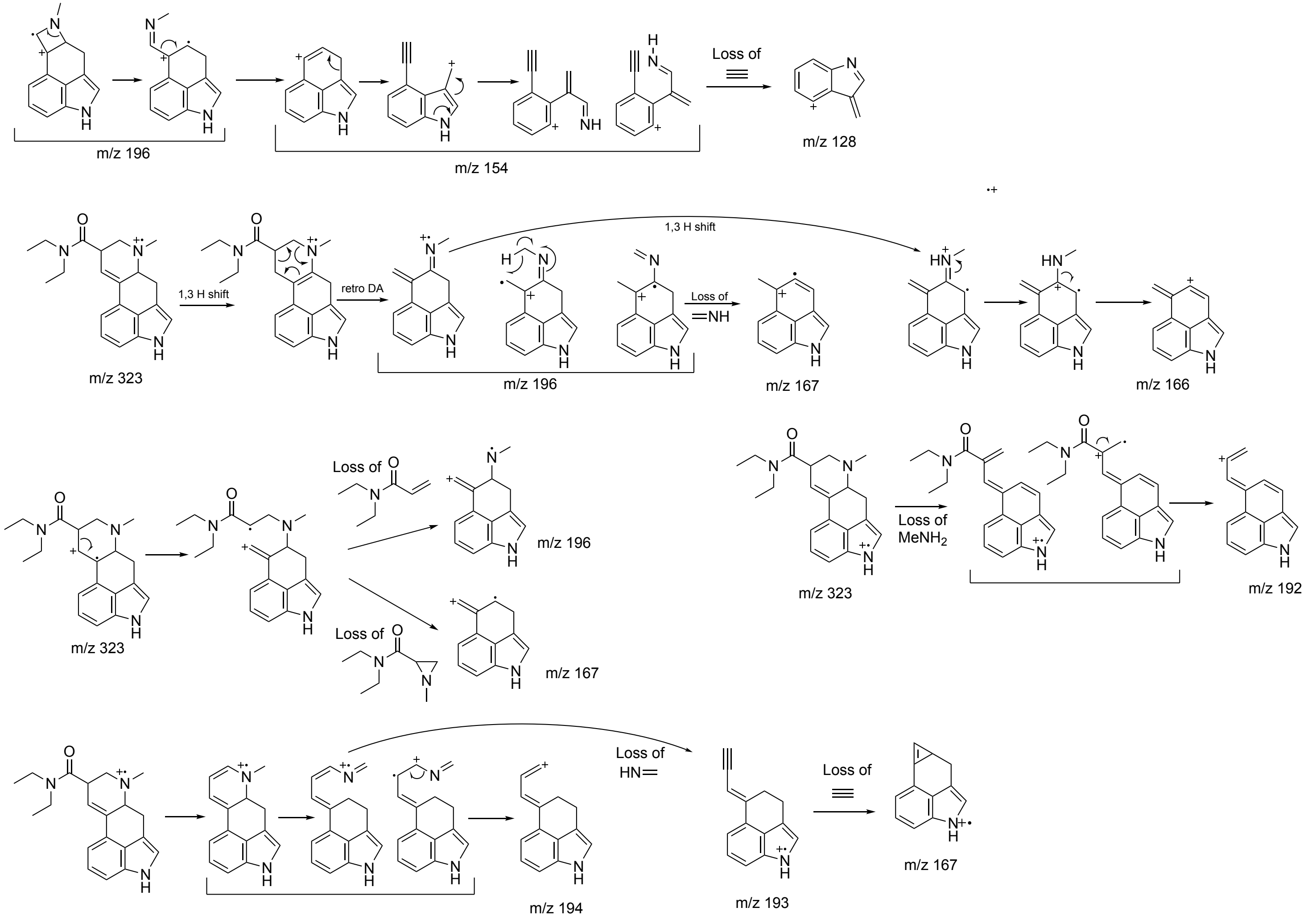

Tentative identification of GC-induced artifacts (GC-MS method 1)

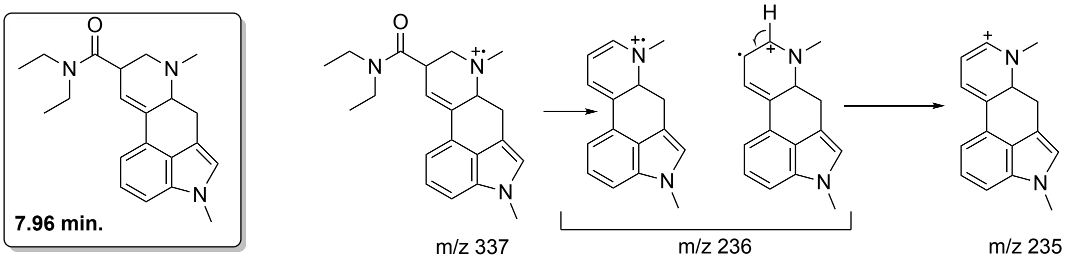

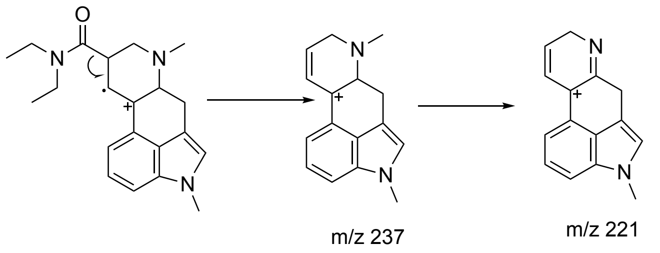

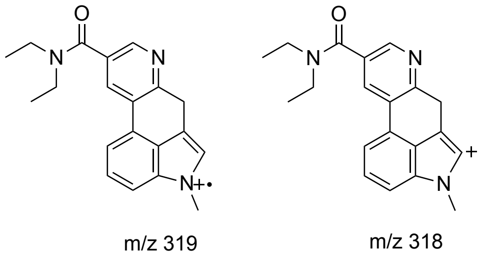

Tentative identification of GC-induced artifacts (GC-MS method 1)

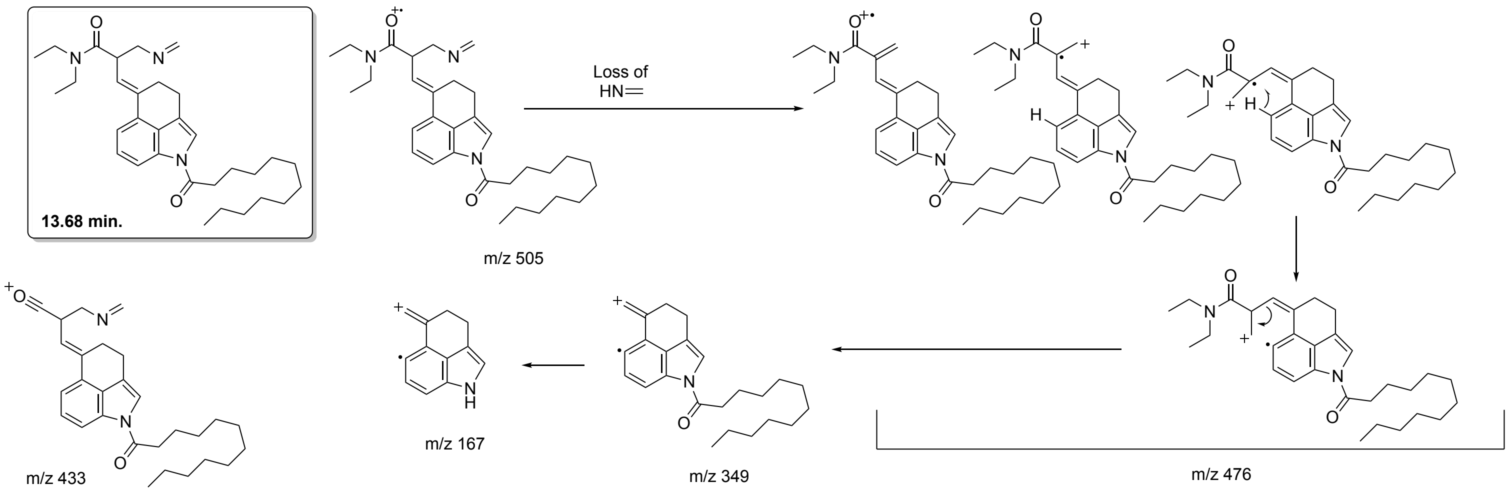

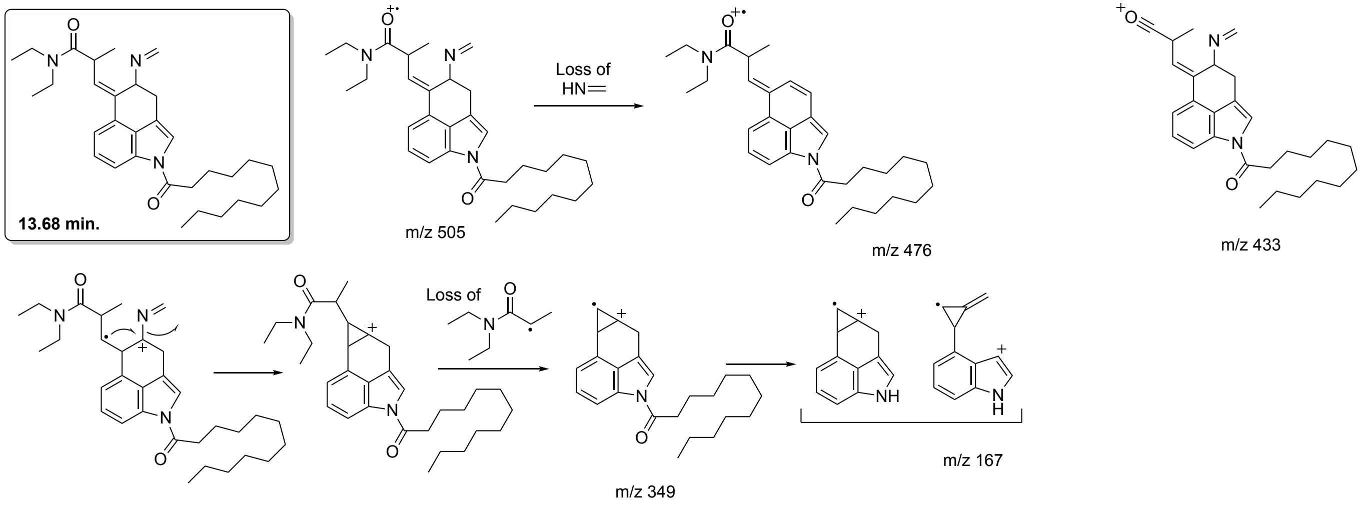

Tentative identification of GC-induced artifacts (GC-MS method 1)

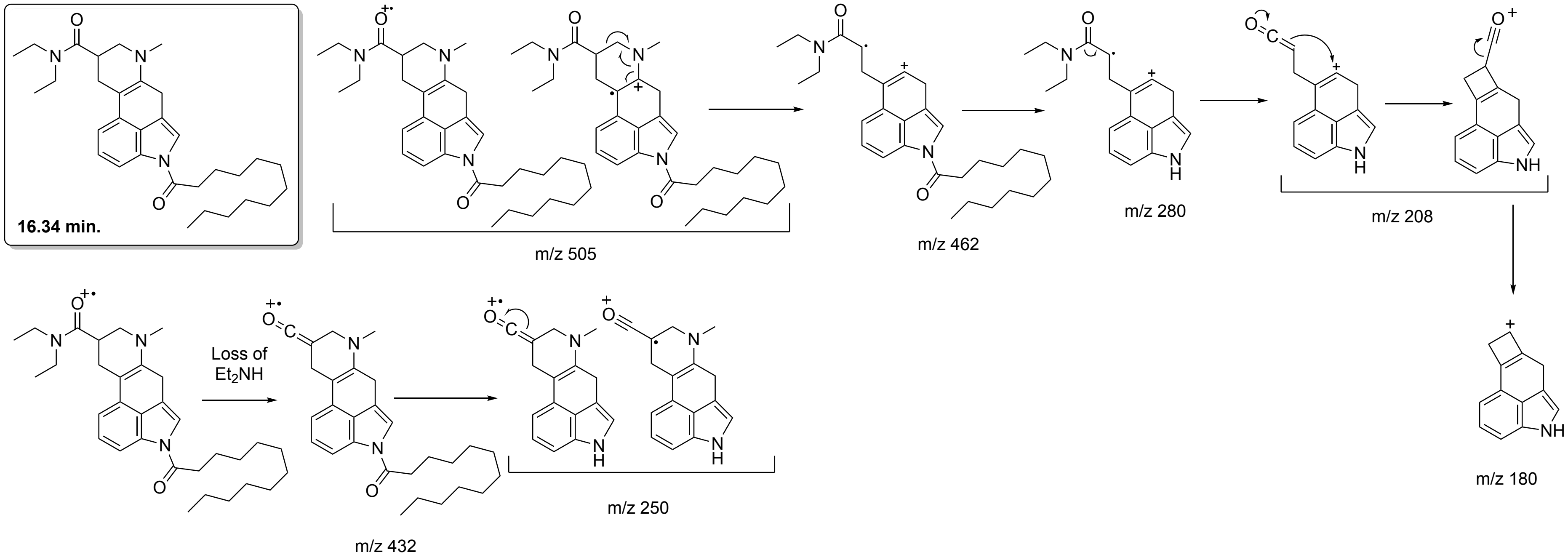

Tentative identification of GC-induced artifacts (GC-MS method 1)

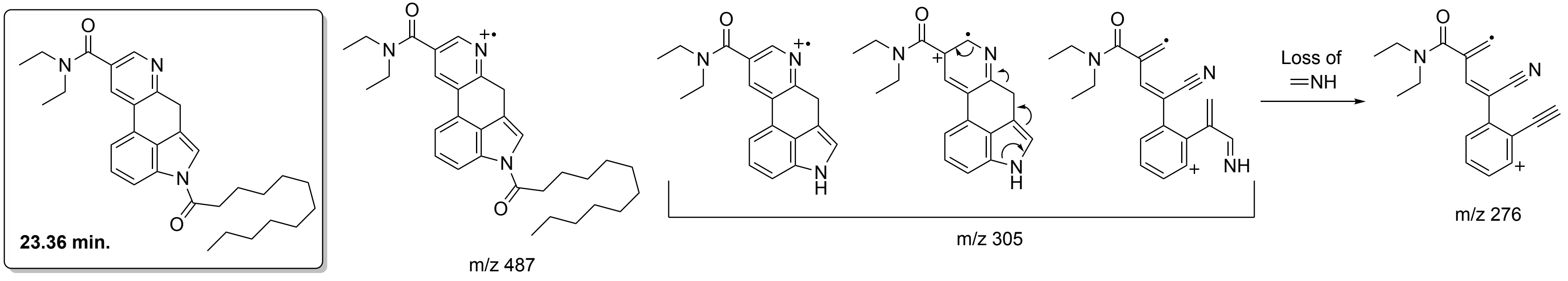

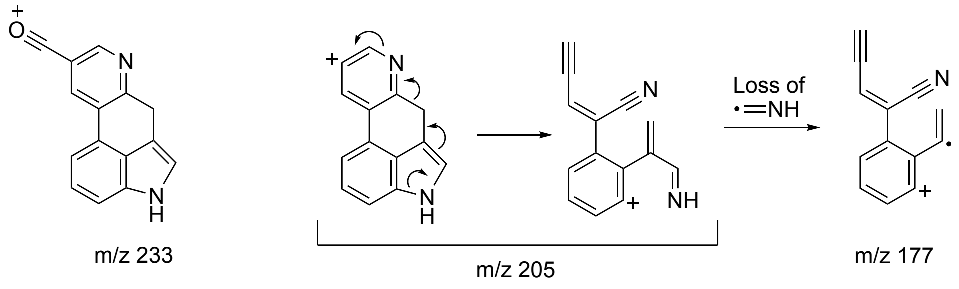

Tentative identification of GC-induced artifacts (GC-MS method 1)

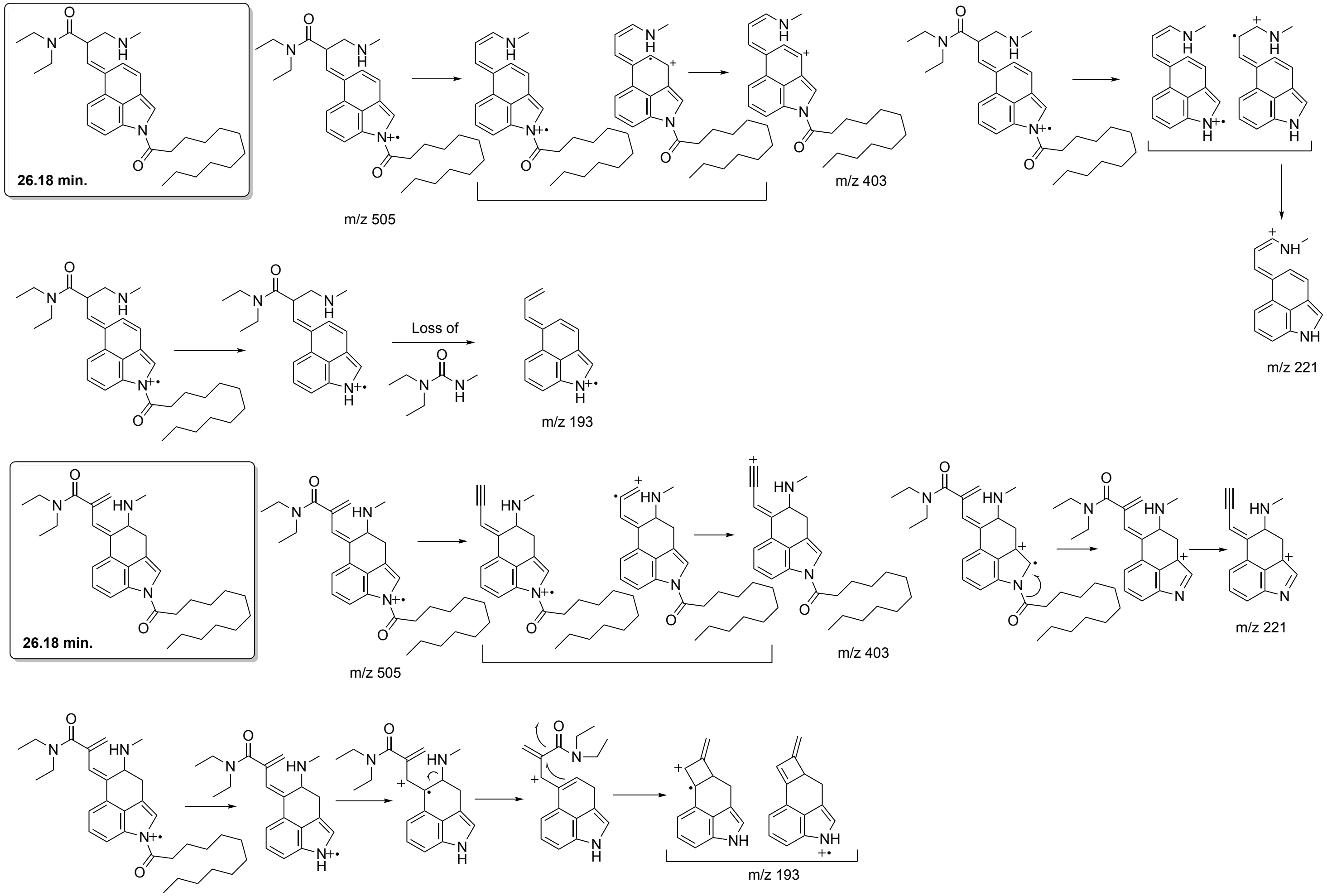

Gas chromatography-mass spectrometry (GC-MS) – method 2

For electron ionization mass spectrometry (EI-MS), a Finnigan TSQ 8000 Evo triple stage quadrupole mass spectrometer coupled to a gas chromatograph (Trace GC 1310, Thermo Electron, Dreieich, Germany) was used and a Triplus RSH (Thermo Scientific) autosampler was employed for sample introduction. Mass spectra were recorded using a 70 eV electron ionization energy. The ion source temperature was set at 175°C and the emission current was 50 µA. For recordings of EI mass spectra, the scan time was 1 s spanning a scan range between *m/z* 29–600 and samples were injected in splitless mode. For the analysis of 1DD-LSD base, the salt (2 mg) was dissolved in 2 mL demineralized water and made alkaline with one drop of NaOH (5% w/w). The solution was extracted with 2 ml diethyl ether, and the ethereal phase was transferred into a new vial and subjected to GC-MS analysis. Separation was achieved using a fused silica capillary DB-1 column (30 m × 0.25 mm, film thickness 0.25 µm). The temperature program consisted of an initial temperature of 80°C, held for 2 min, followed by a ramp to 340°C at 15°C/min. The final temperature was held for 20 min. The injector temperature was 280°C. The transfer line temperature was set at 280°C and the carrier gas was helium in constant flow mode at a flow rate of 1.2 mL/min. Mass spectra were treated as a sum of 6 spectra (AV:6), from 17.99 to 18.07 min. Background spectra were subtracted twice: from 17.57 and 17.72 min before and from 18.44 to 18.57 min after the peak in EIC mode. RI values could not be determined under these conditions when using a paraffin mixture at oven temperatures up to 340°C (RI > 4000).

GC-MS data (method 2)

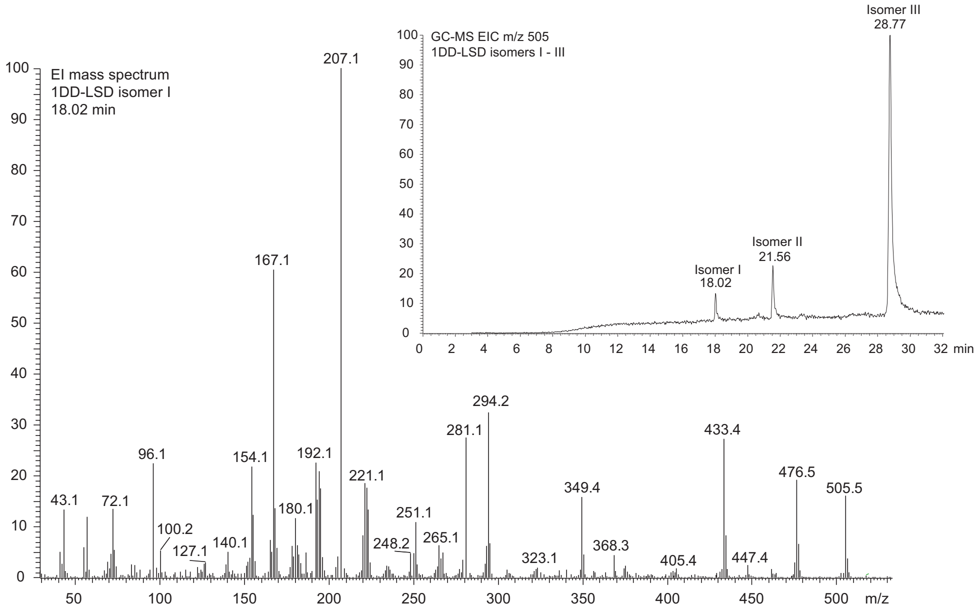

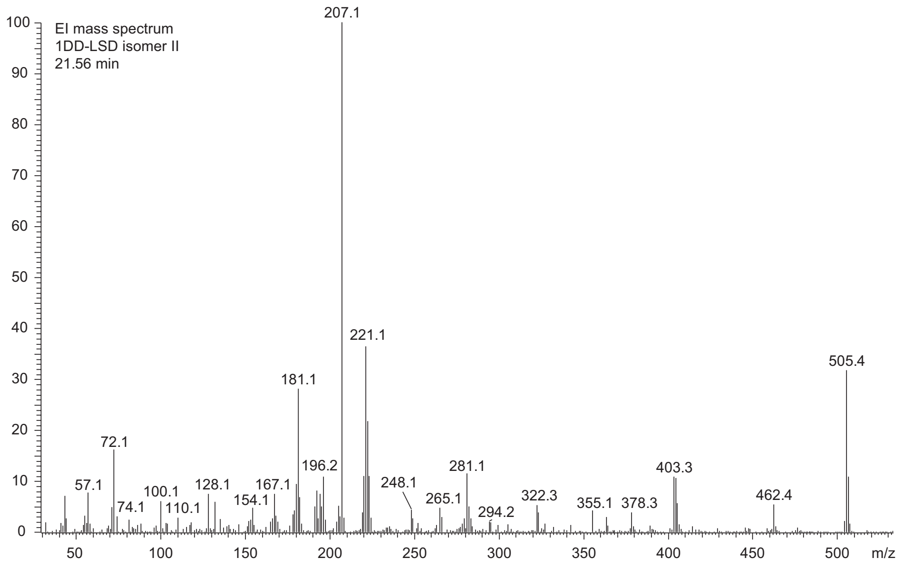

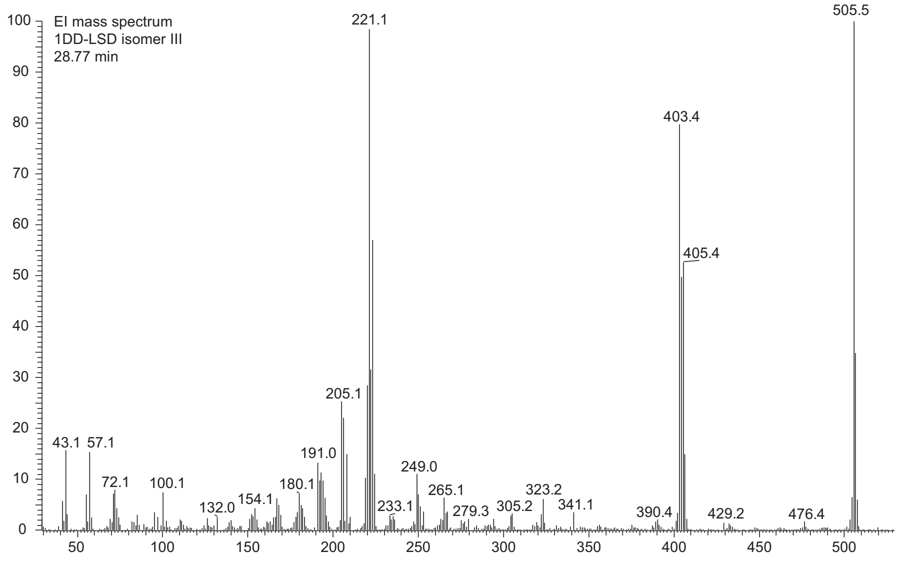

Proposed ESI-QTOF-MS/MS fragmentation pathways for 1DD-LSD

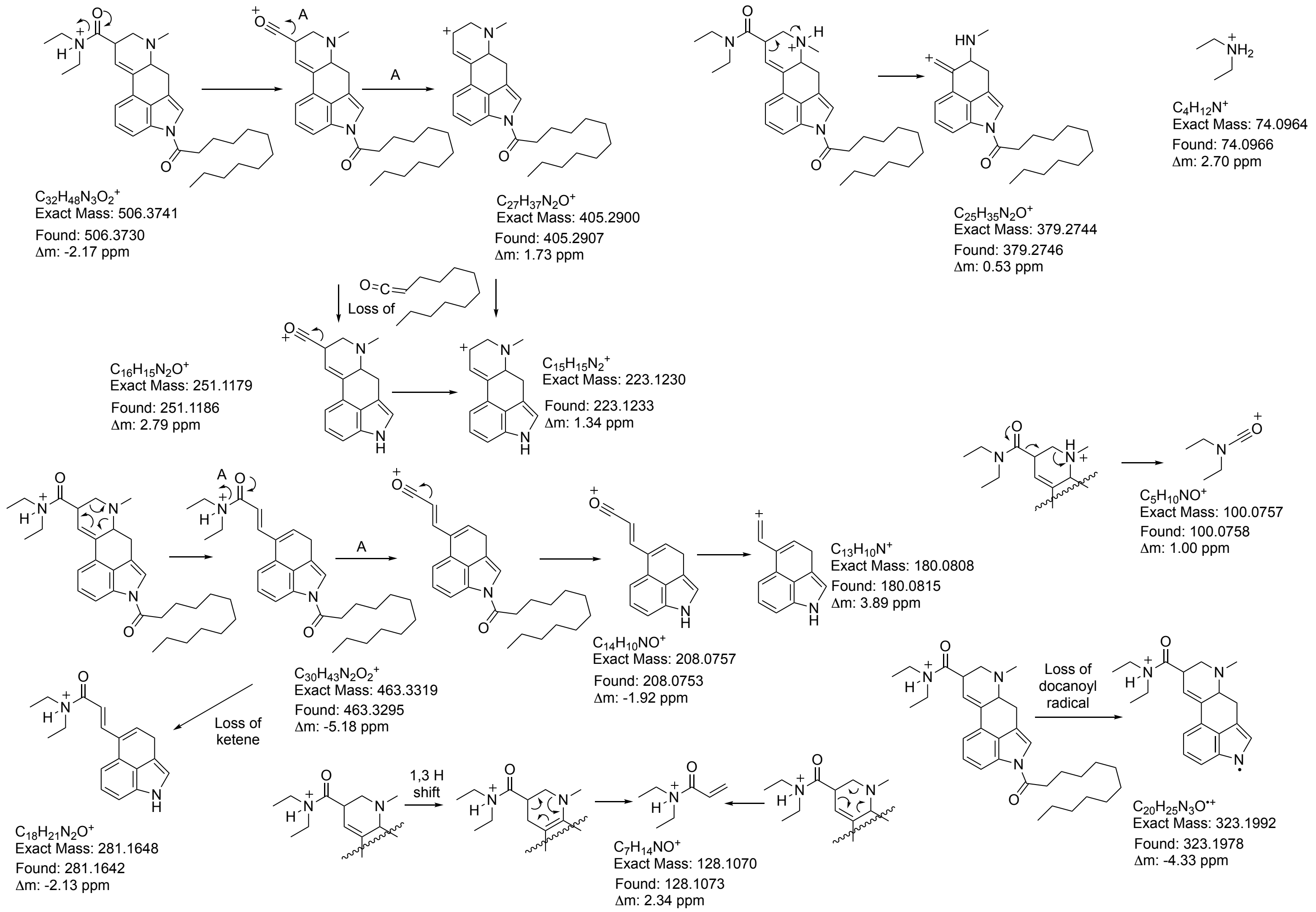

LC-ESI-linear ion trap-MS/MS

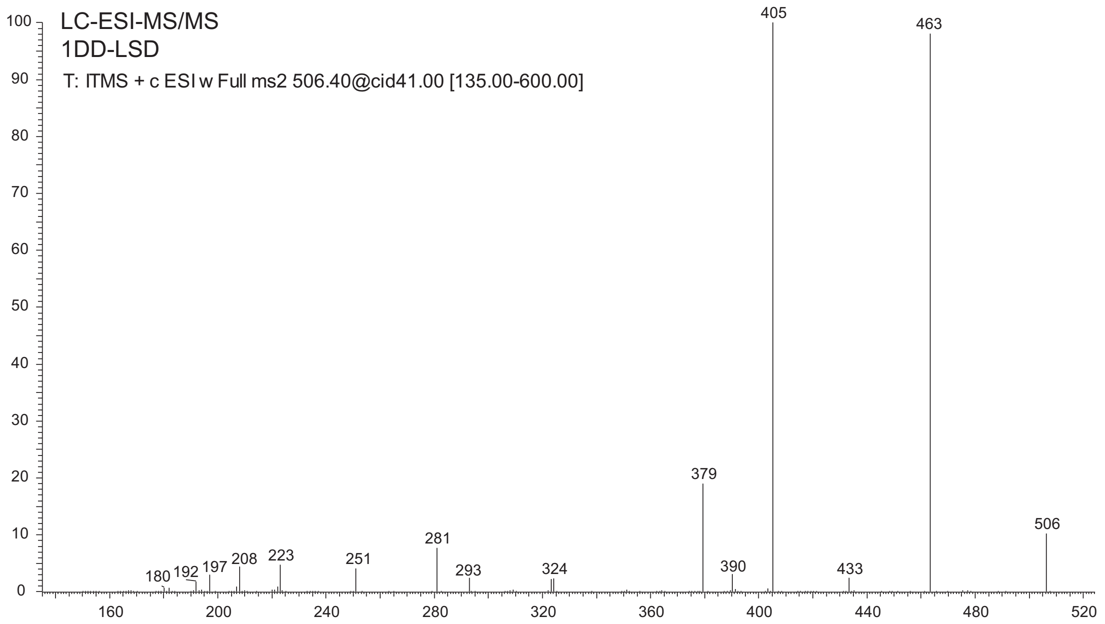

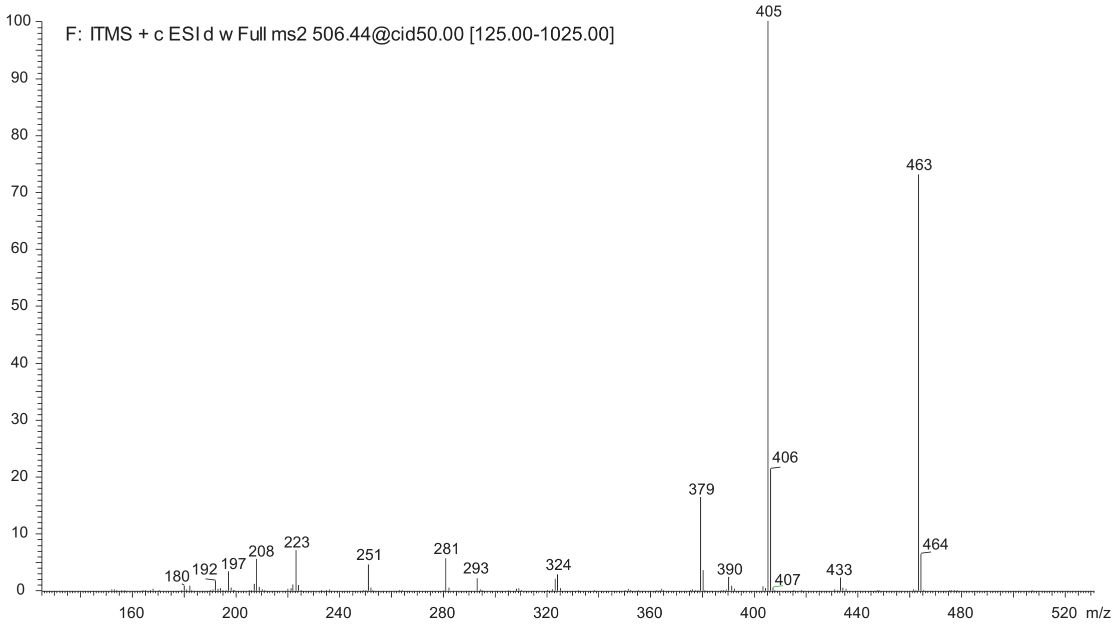

LC-ESI-linear ion trap-MS

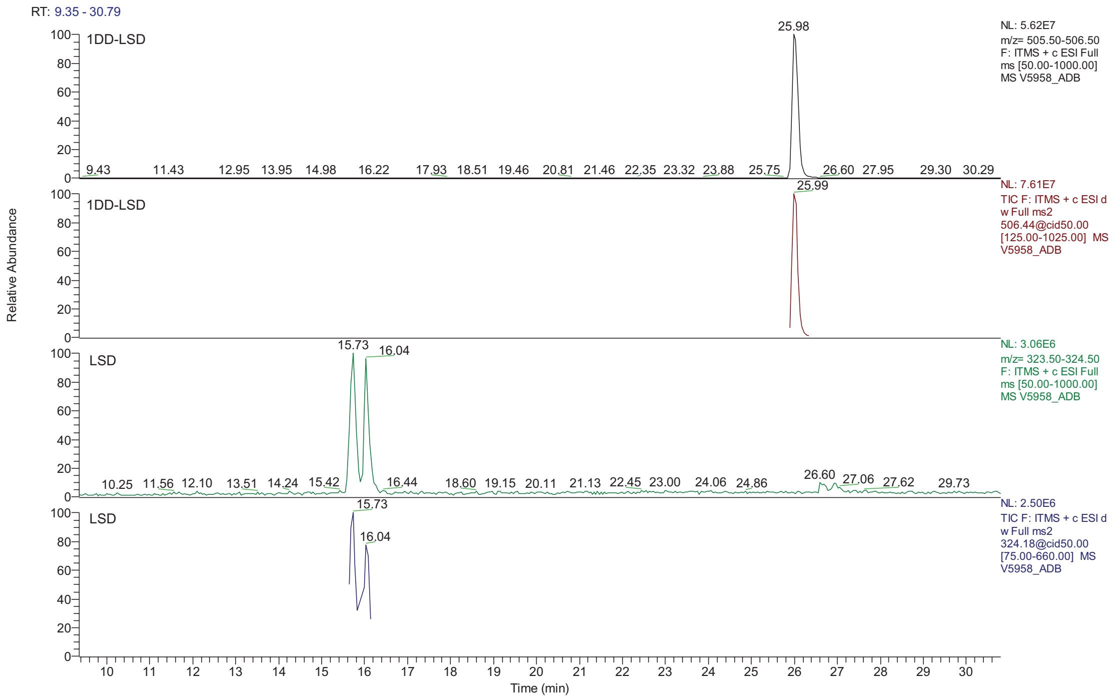

Attenuated total reflection-infrared spectroscopy (ATR-IR)

The IR spectrum of the powdered 1DD-LSD tartrate (3:2) was recorded on a Perkin Elmer Spectrum 100 FT–IR with Universal ATR sampling accessory (Perkin Elmer, Waltham, MA, USA). The wavelength resolution was set to 2 cm^{-1}. IR spectra were collected in a range of 650–4000 cm^{-1} with 16 scans per spectrum. The IR data were processed using Spectrum Perkin Elmer Version 6.3.4 Software (Perkin Elmer, Waltham, MA, USA).

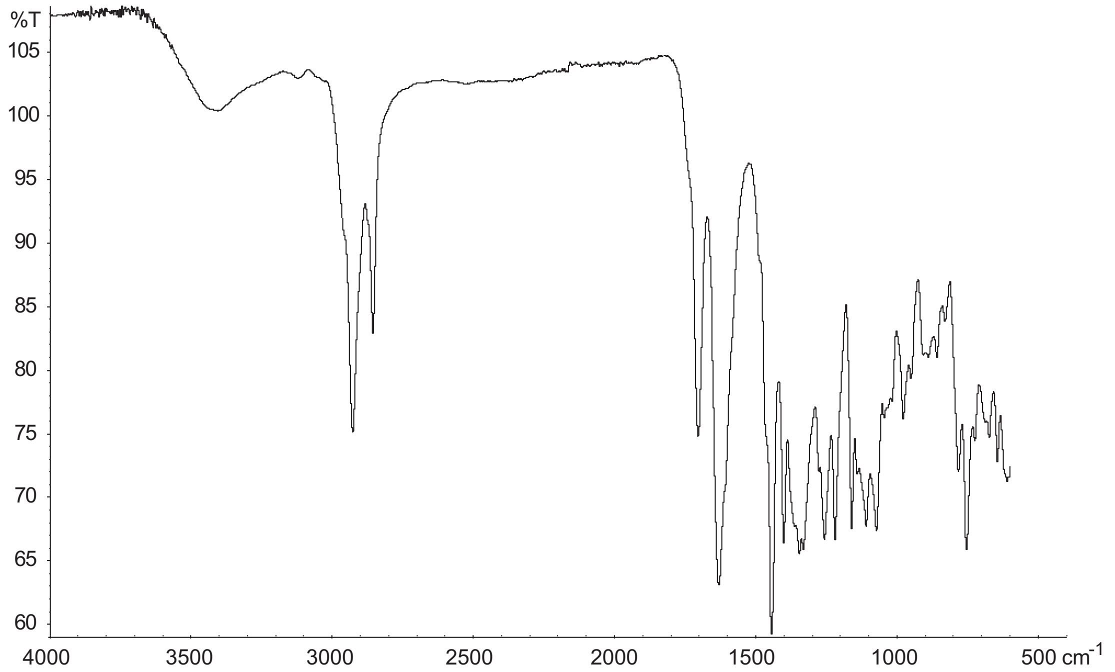

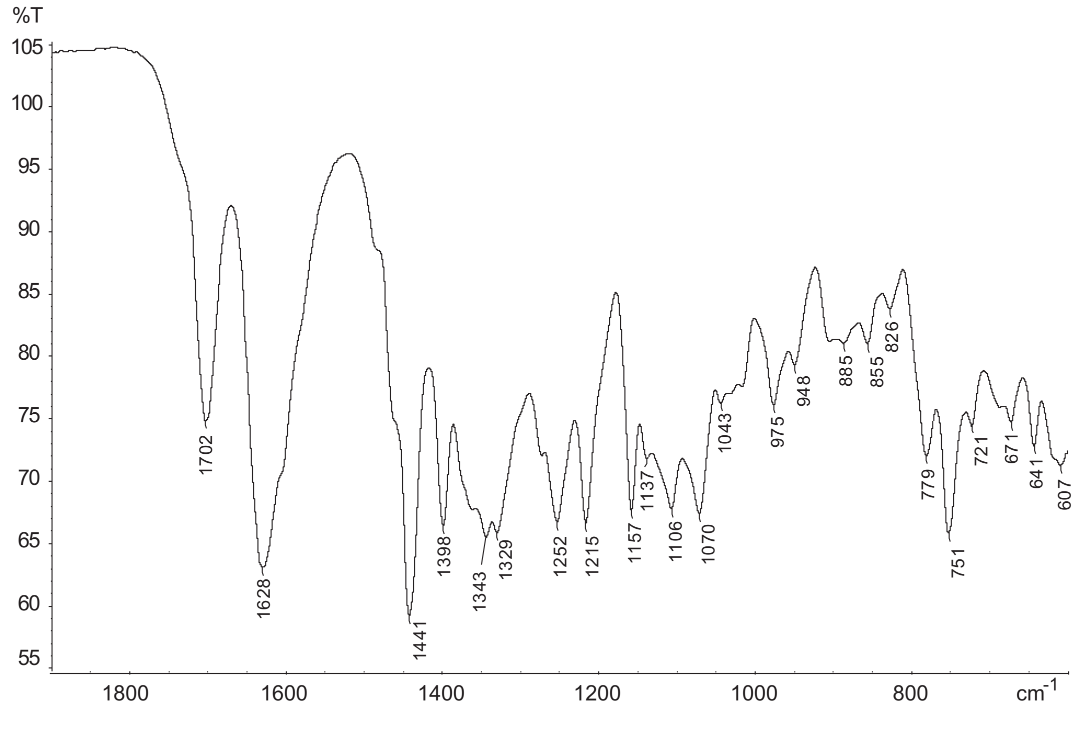

1DD-LSD tartrate (3:2)
1H NMR (600 MHz)
DMSO-d_6

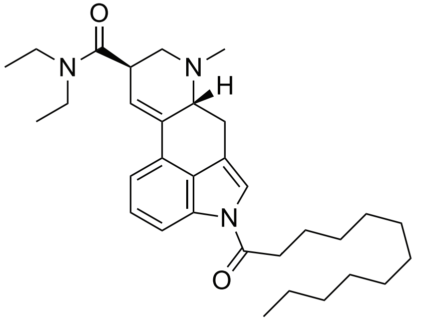

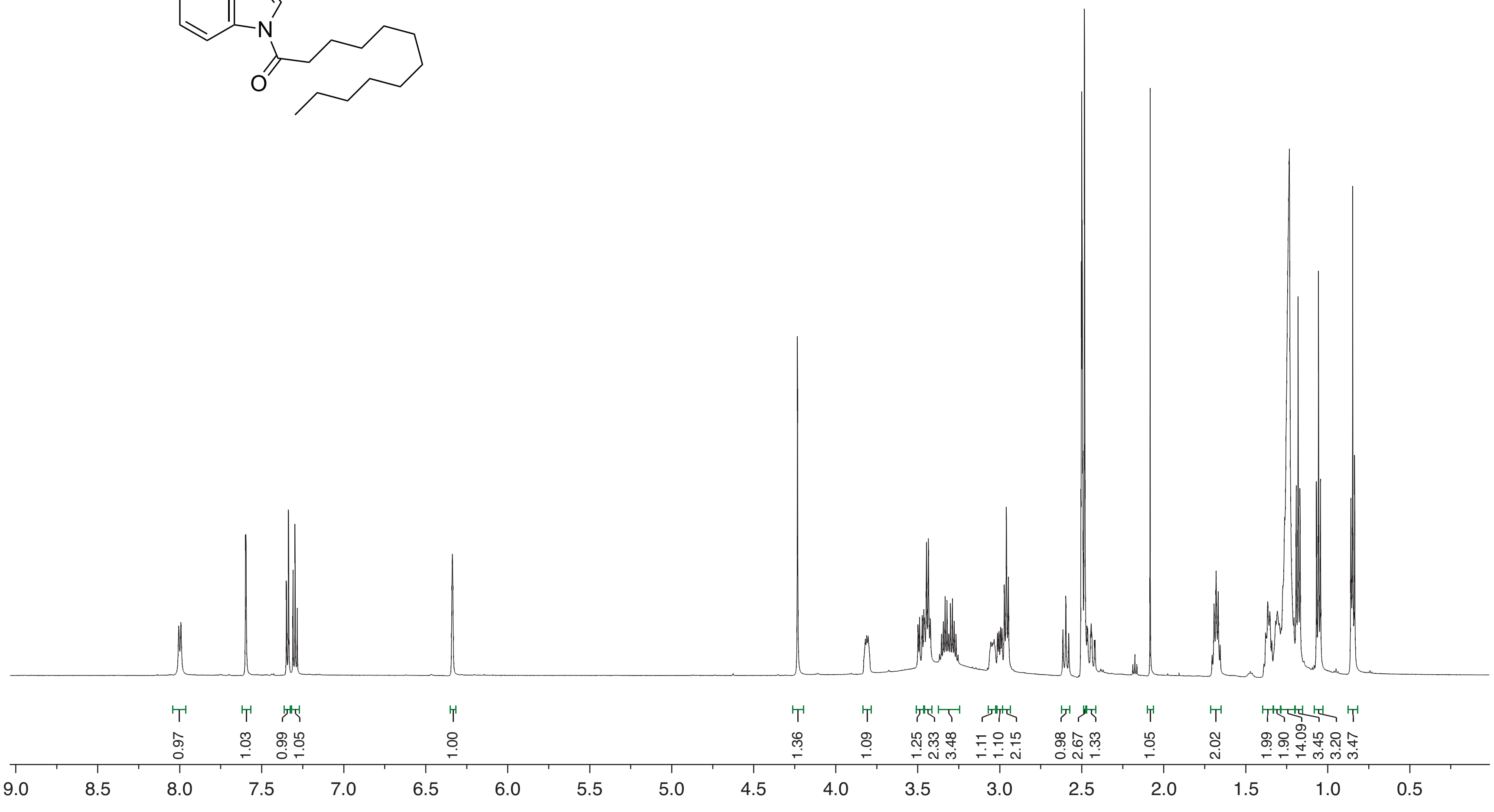

1DD-LSD tartrate (3:2)
1H NMR (600 MHz)
DMSO-d_6

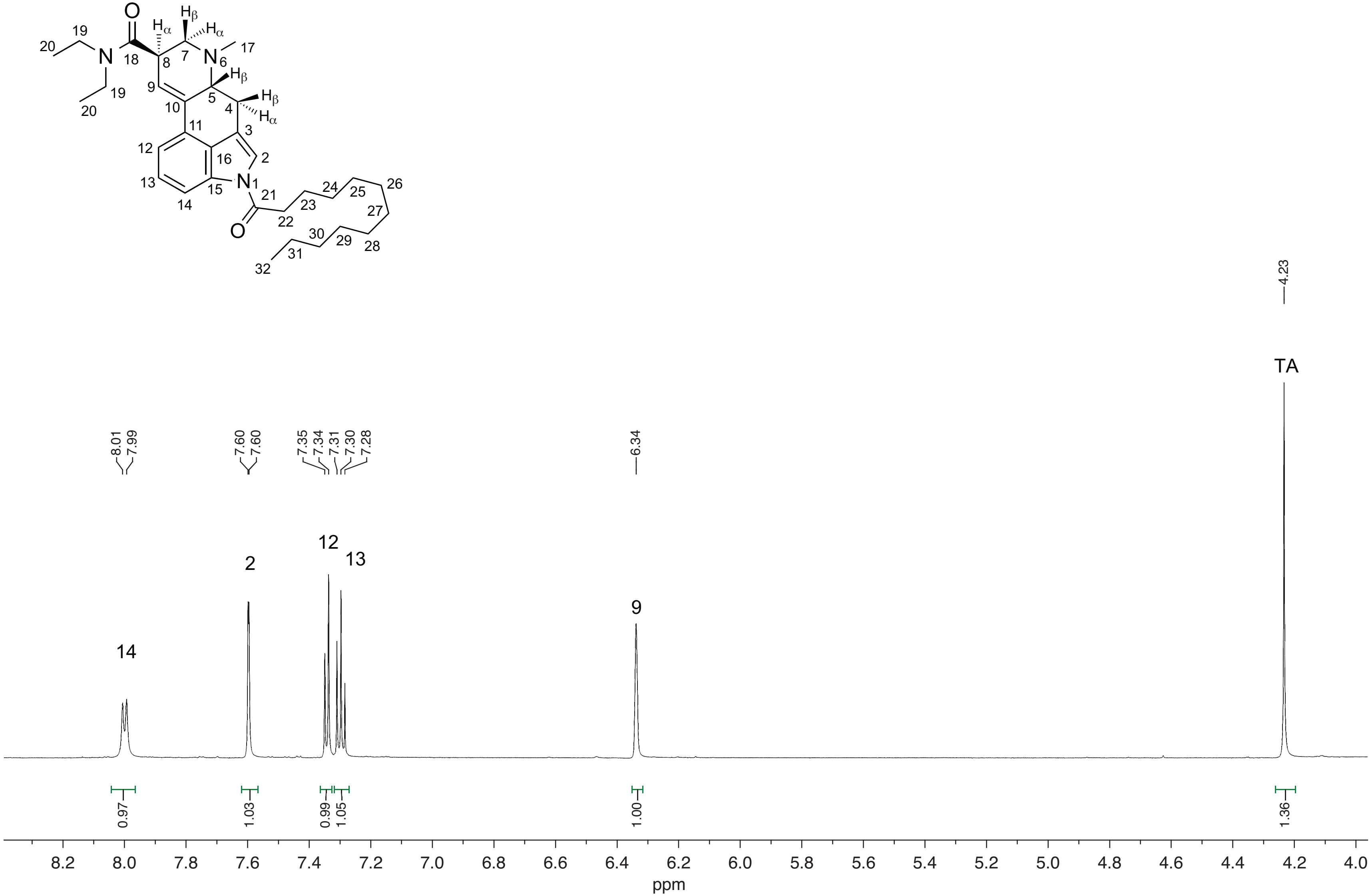

TA: tartaric acid

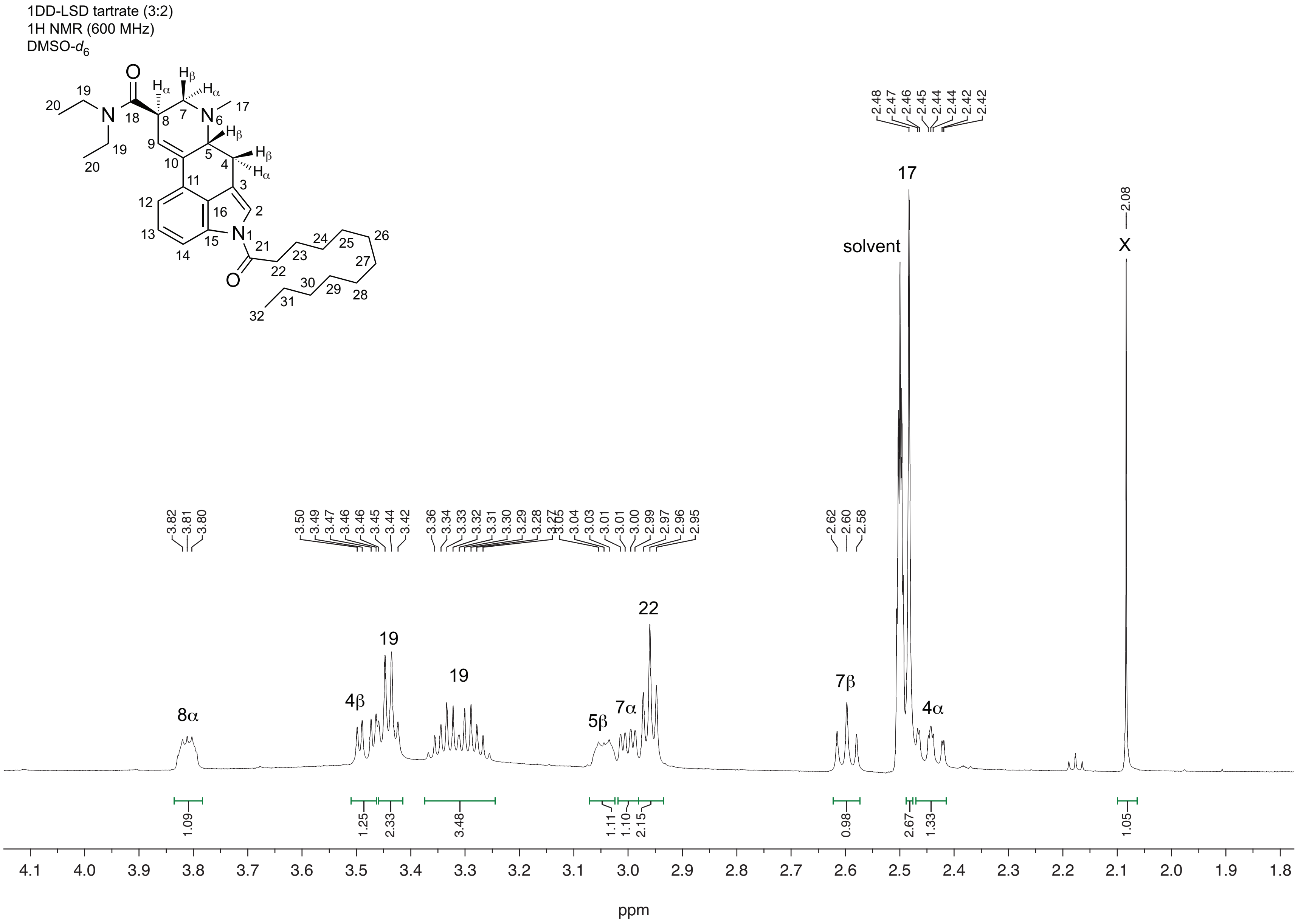
1DD-LSD tartrate (3:2)
1H NMR (600 MHz)
DMSO-d_6
8α
4β
19
19
5β
7α
22
7β
solvent
17
4α
X
ppm

1DD-LSD tartrate (3:2)
1H NMR (600 MHz)
DMSO-d_6

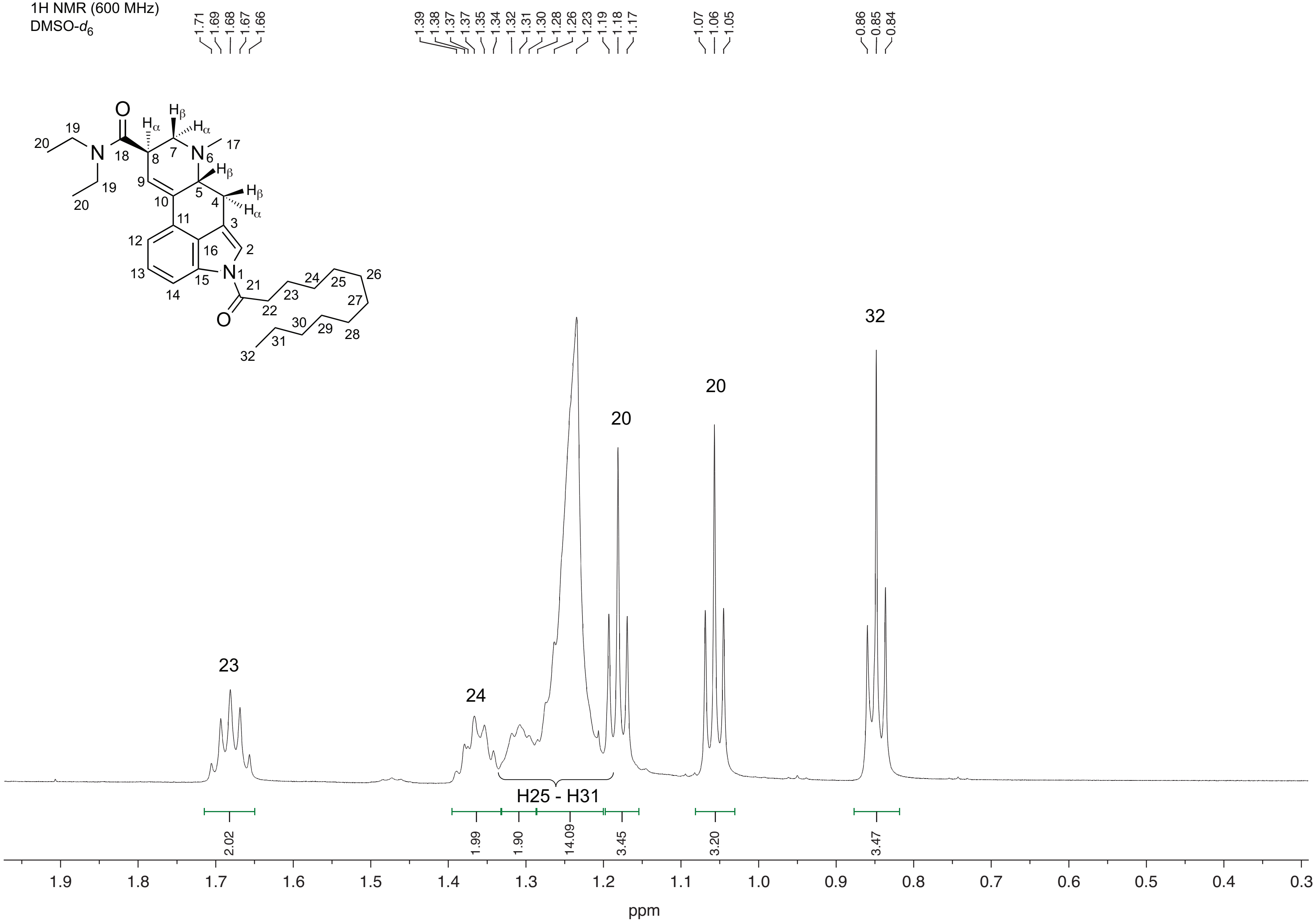

1DD-LSD tartrate (3:2)
COSY (600 MHz)
DMSO-d_6

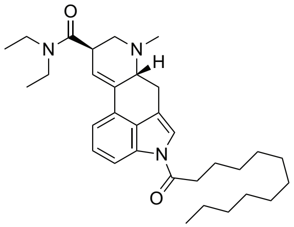

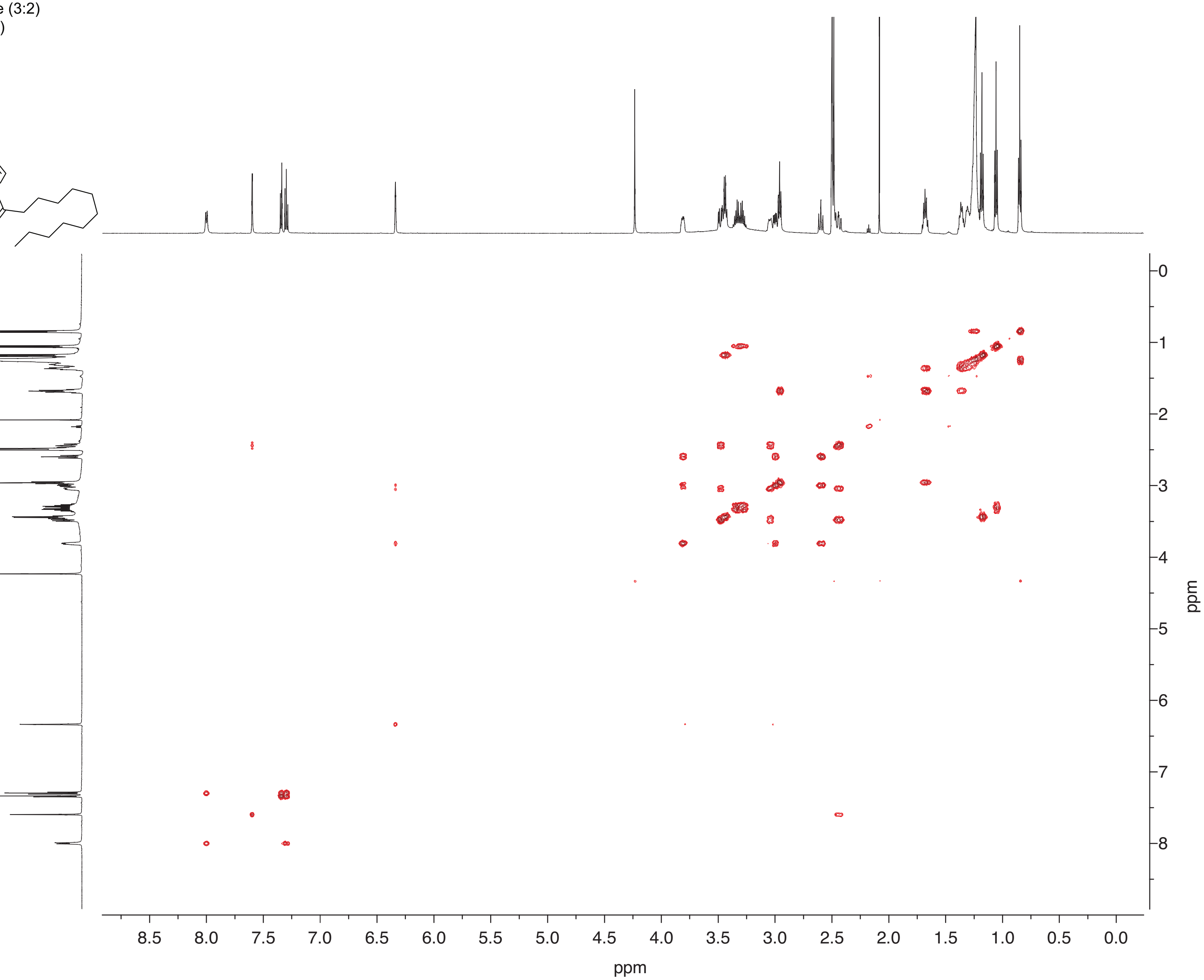

1DD-LSD tartrate (3:2)
COSY (600 MHz)
DMSO-d_6

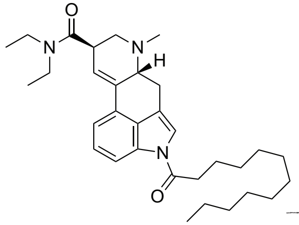

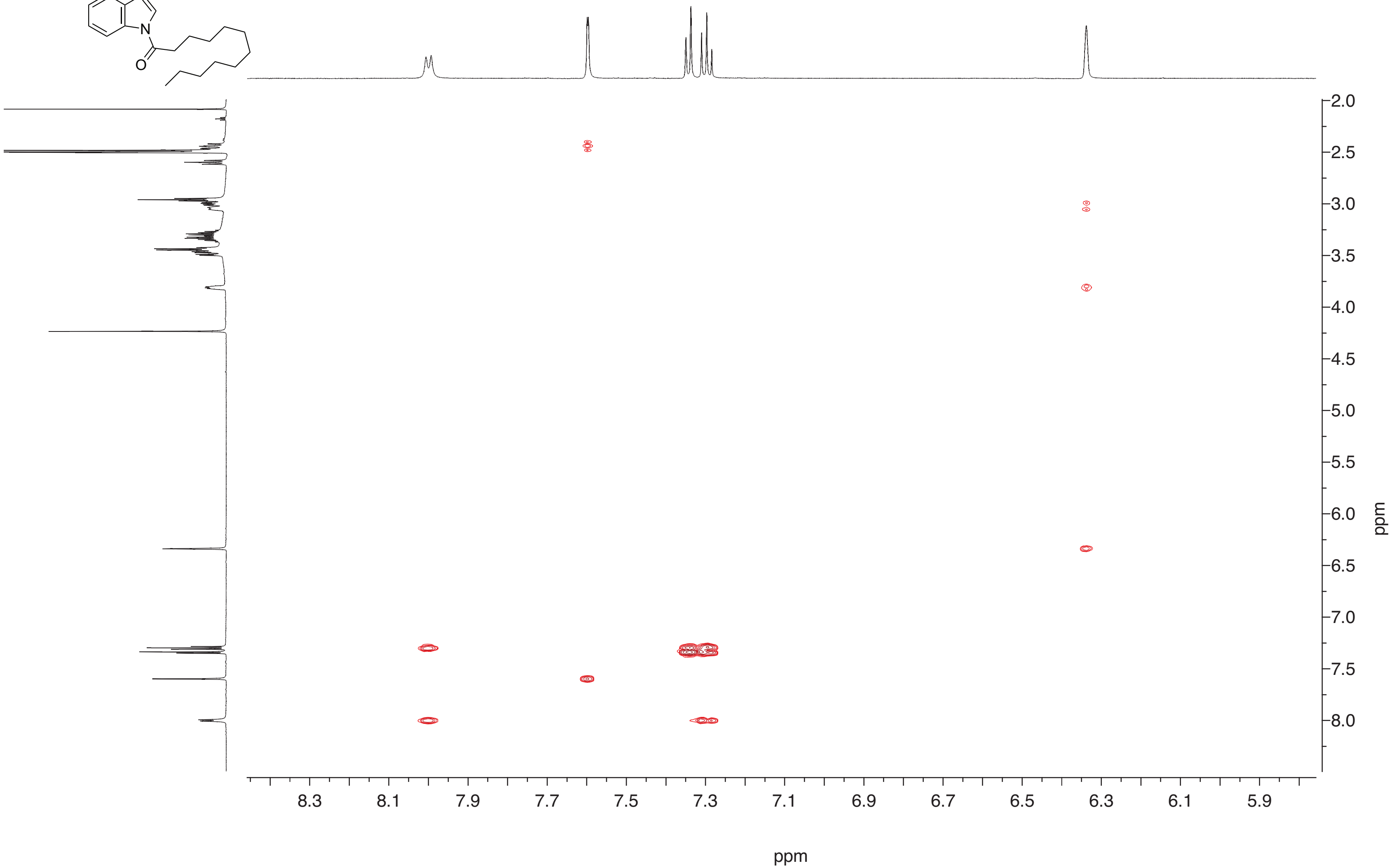

1DD-LSD tartrate (3:2)
COSY (600 MHz)
DMSO-d_6

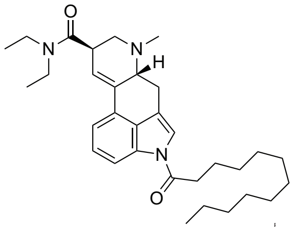

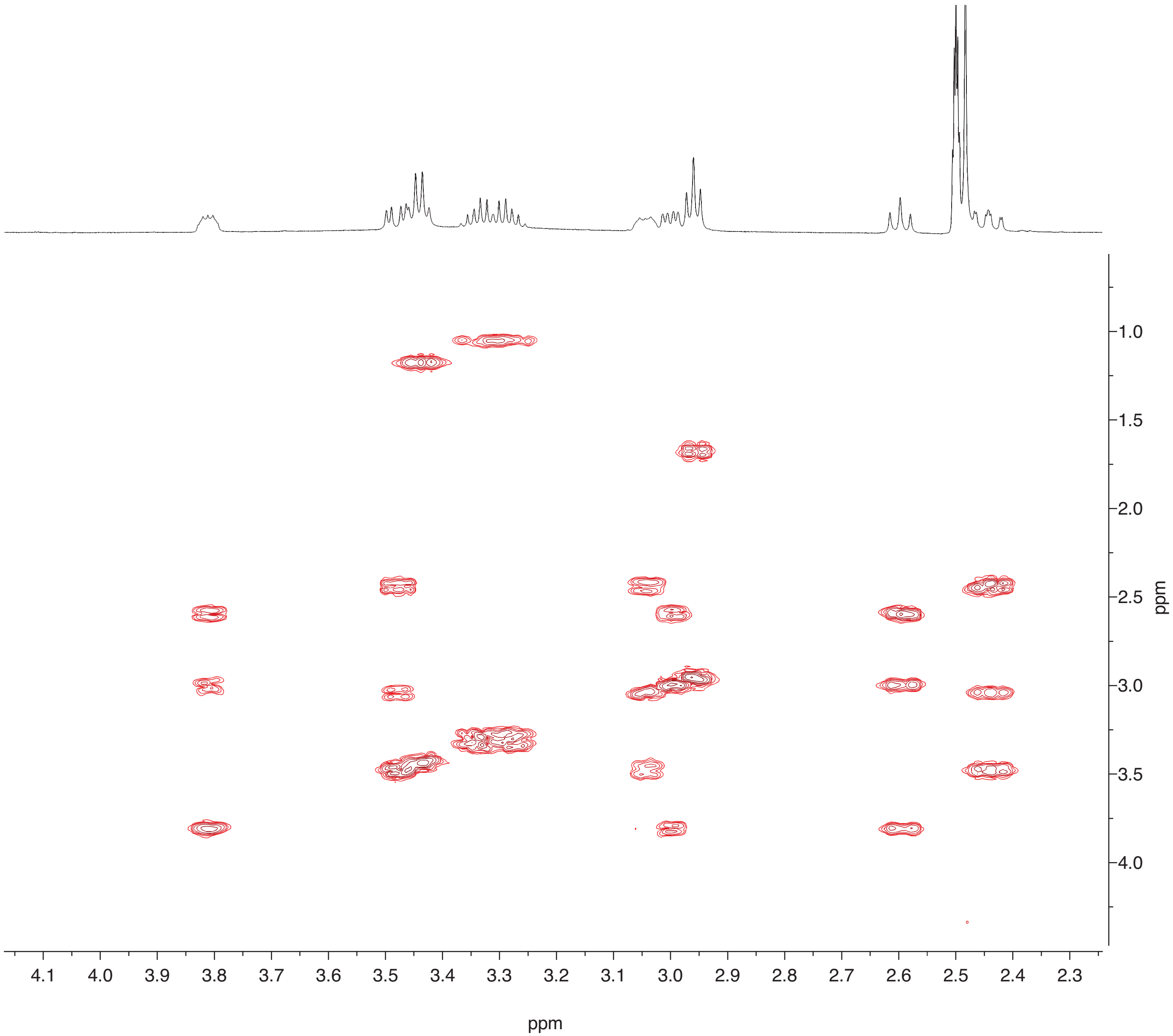

1DD-LSD tartrate (3:2)
COSY (600 MHz)
DMSO-d_6

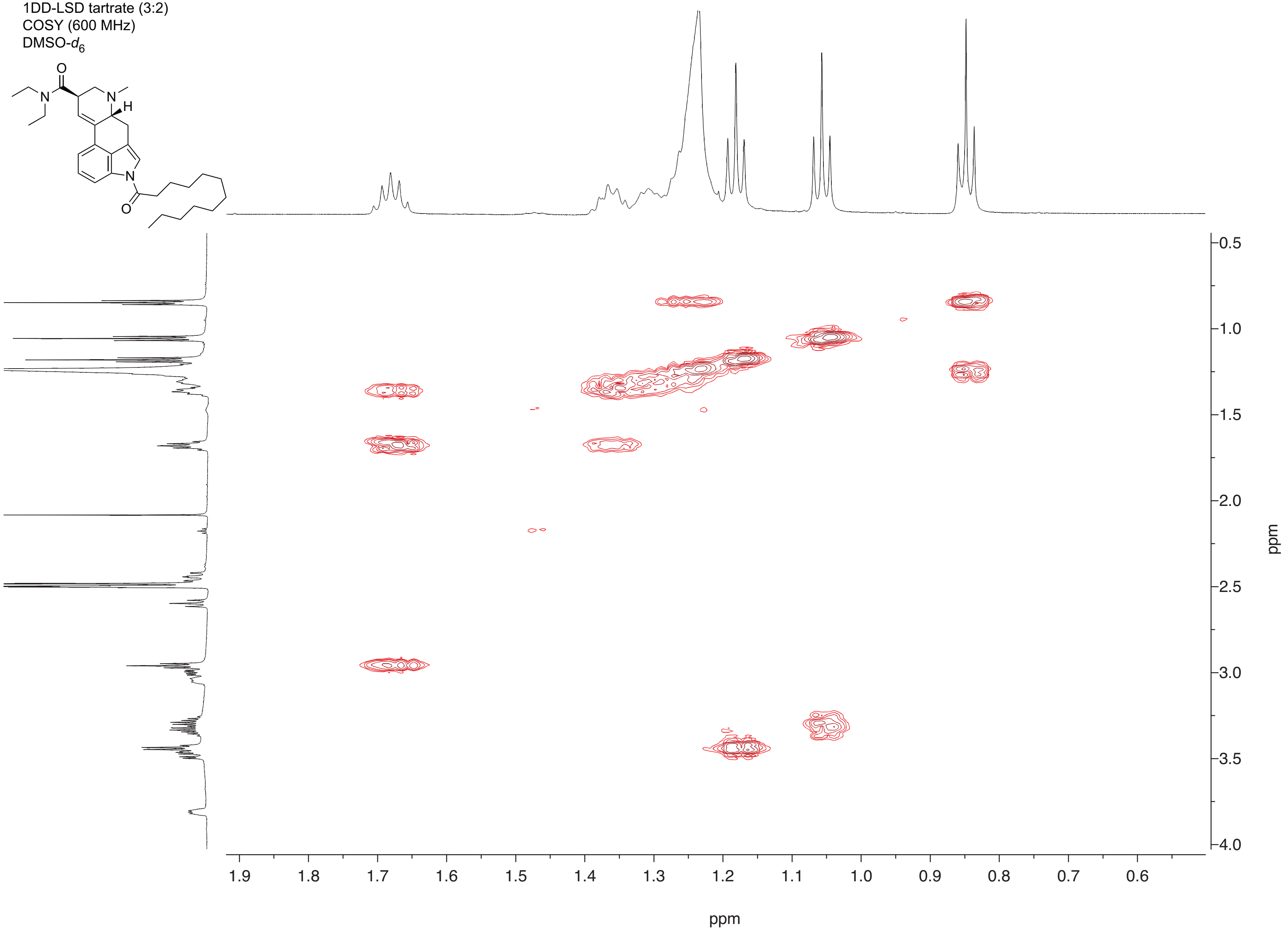

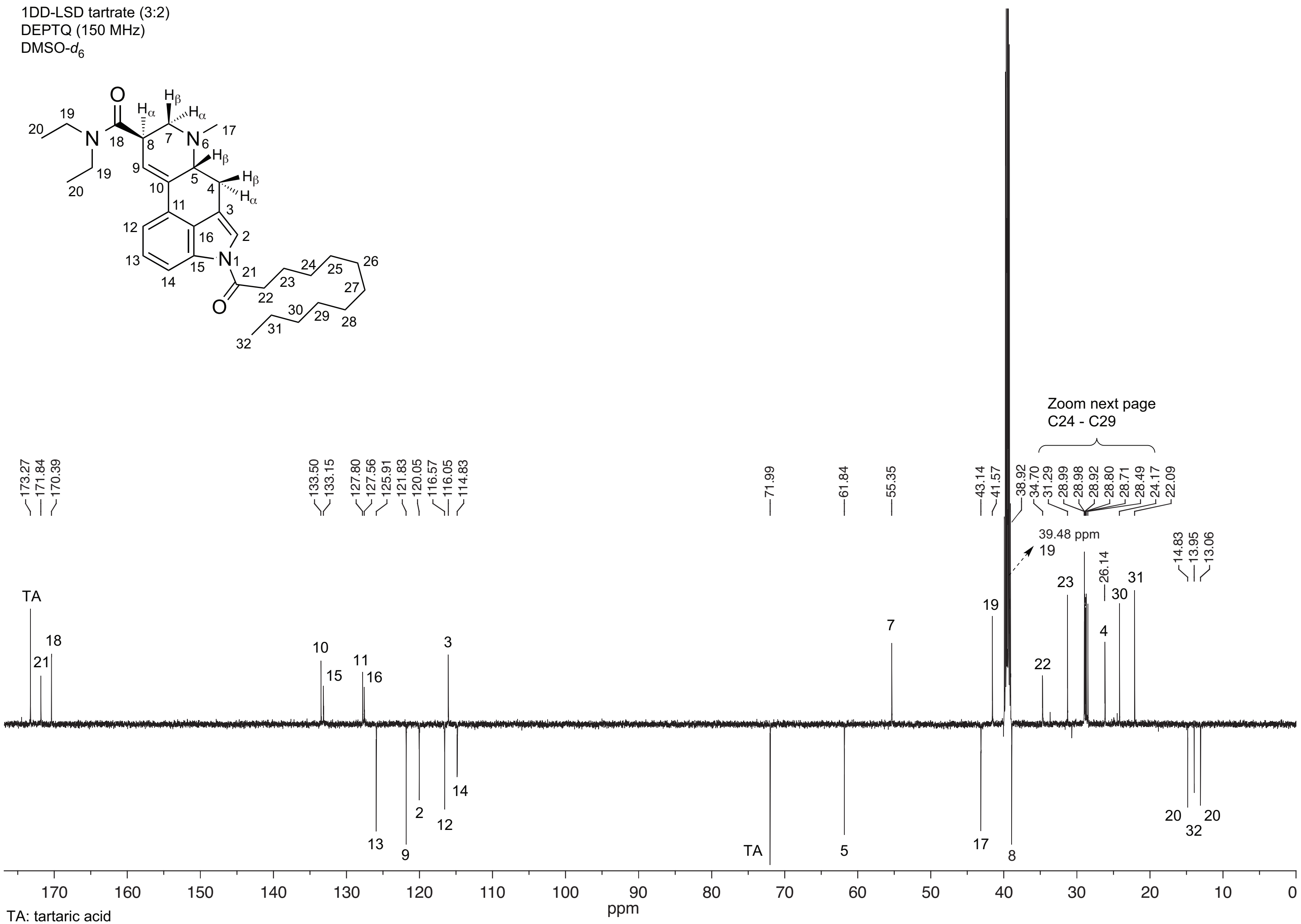

TA: tartaric acid

1DD-LSD tartrate (3:2)
DEPTQ (150 MHz)
DMSO-d_6

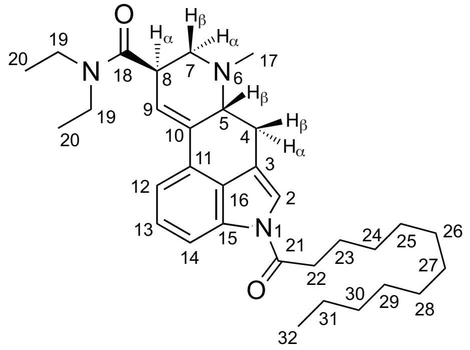

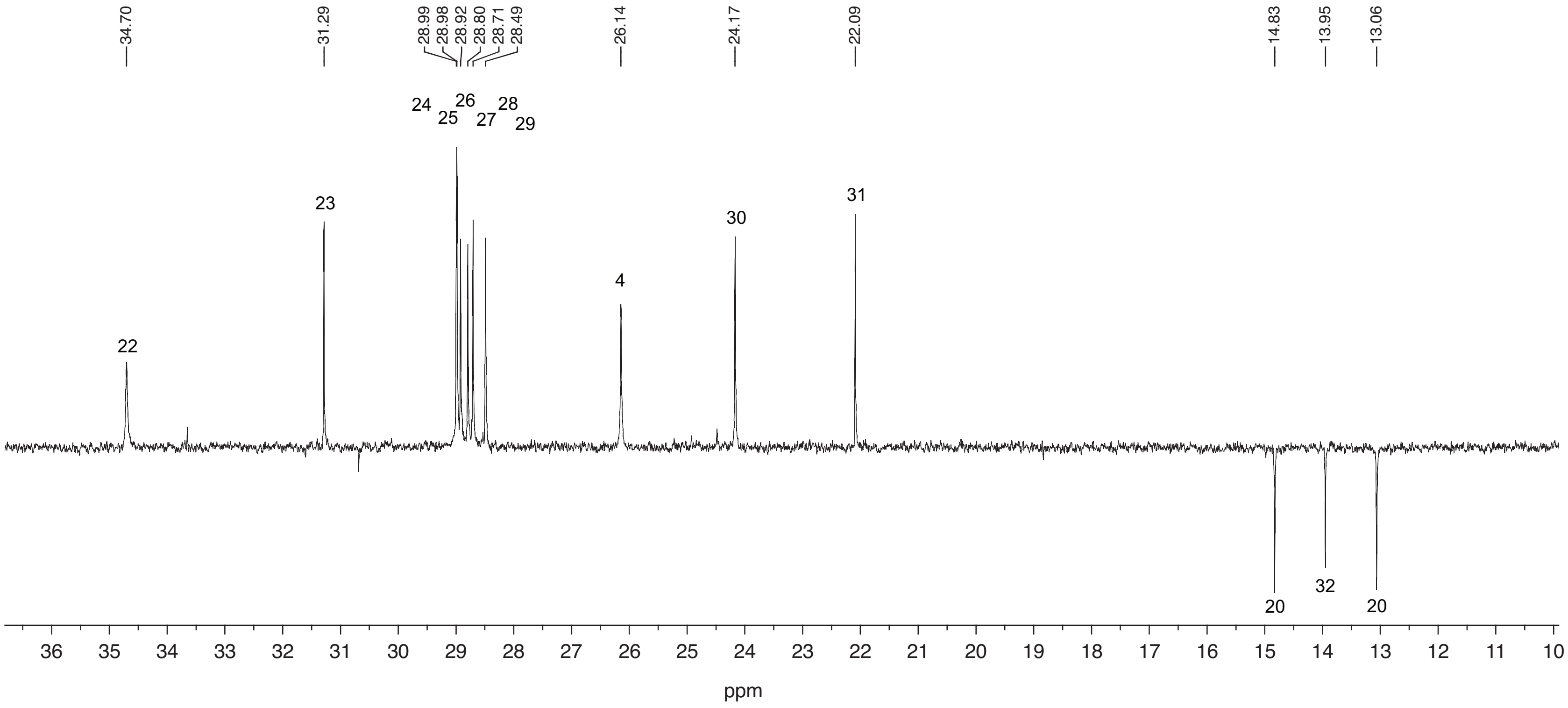

1DD-LSD tartrate (3:2)
HSQC (600/150 MHz)
DMSO-d_6

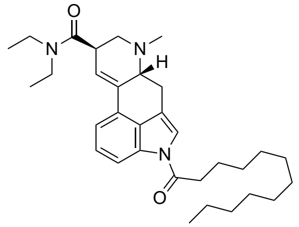

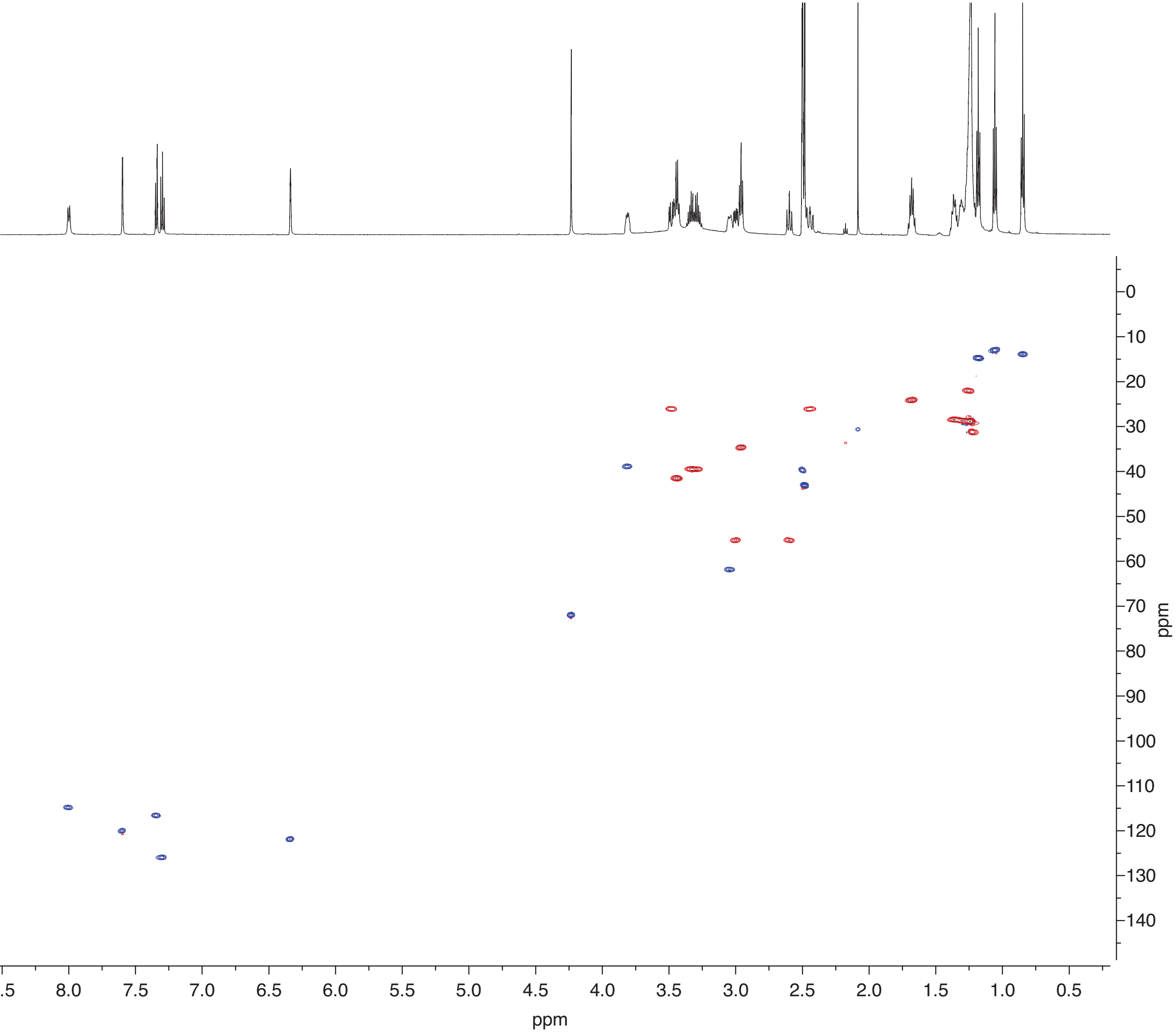

1DD-LSD tartrate (3:2)
HSQC (600/150 MHz)
DMSO-d_6

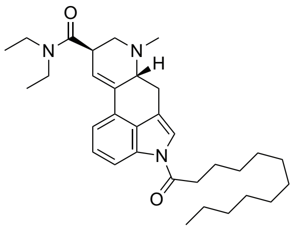

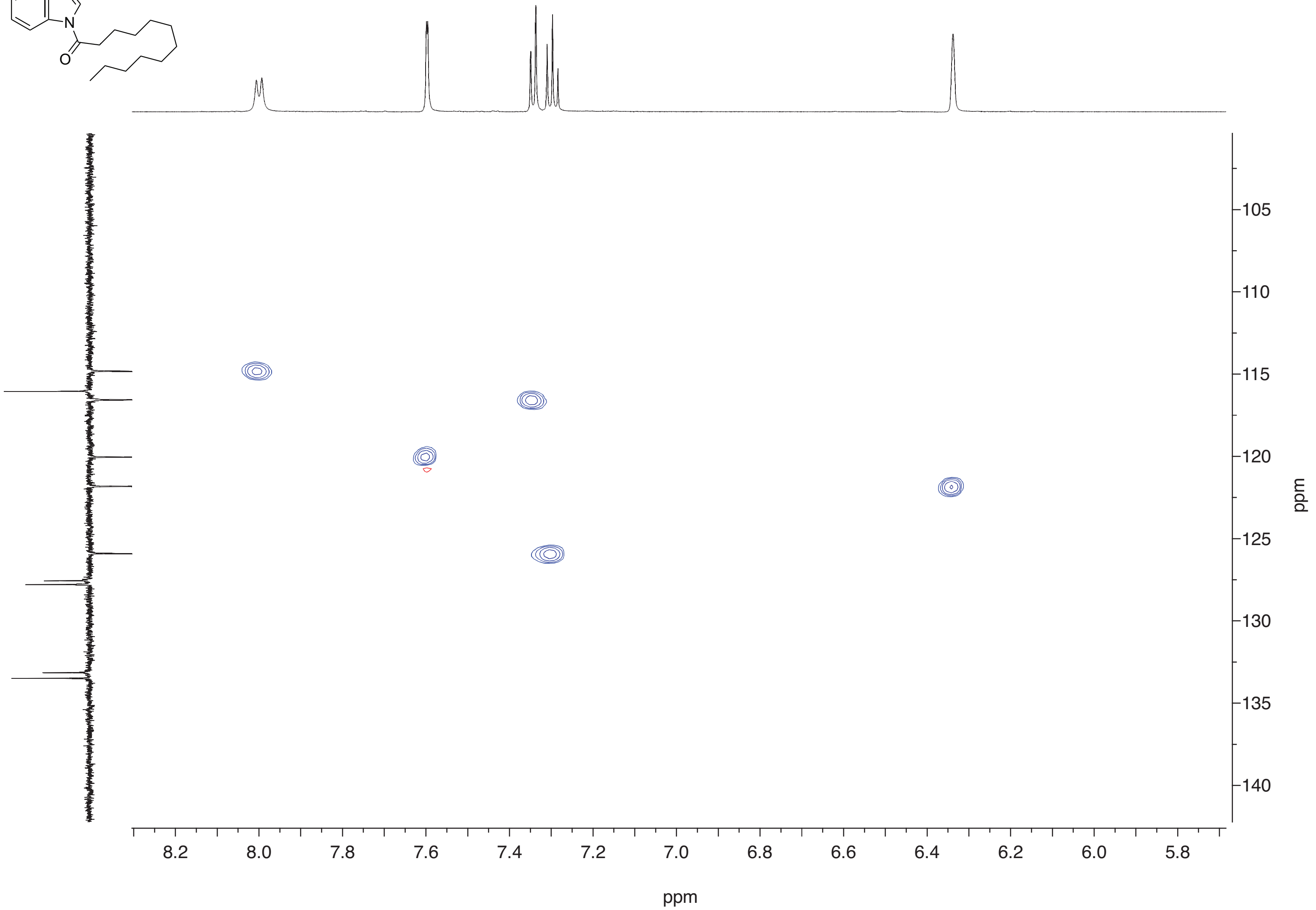

1DD-LSD tartrate (3:2)
HSQC (600/150 MHz)
DMSO-d_6

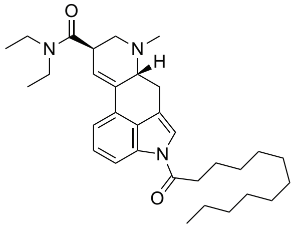

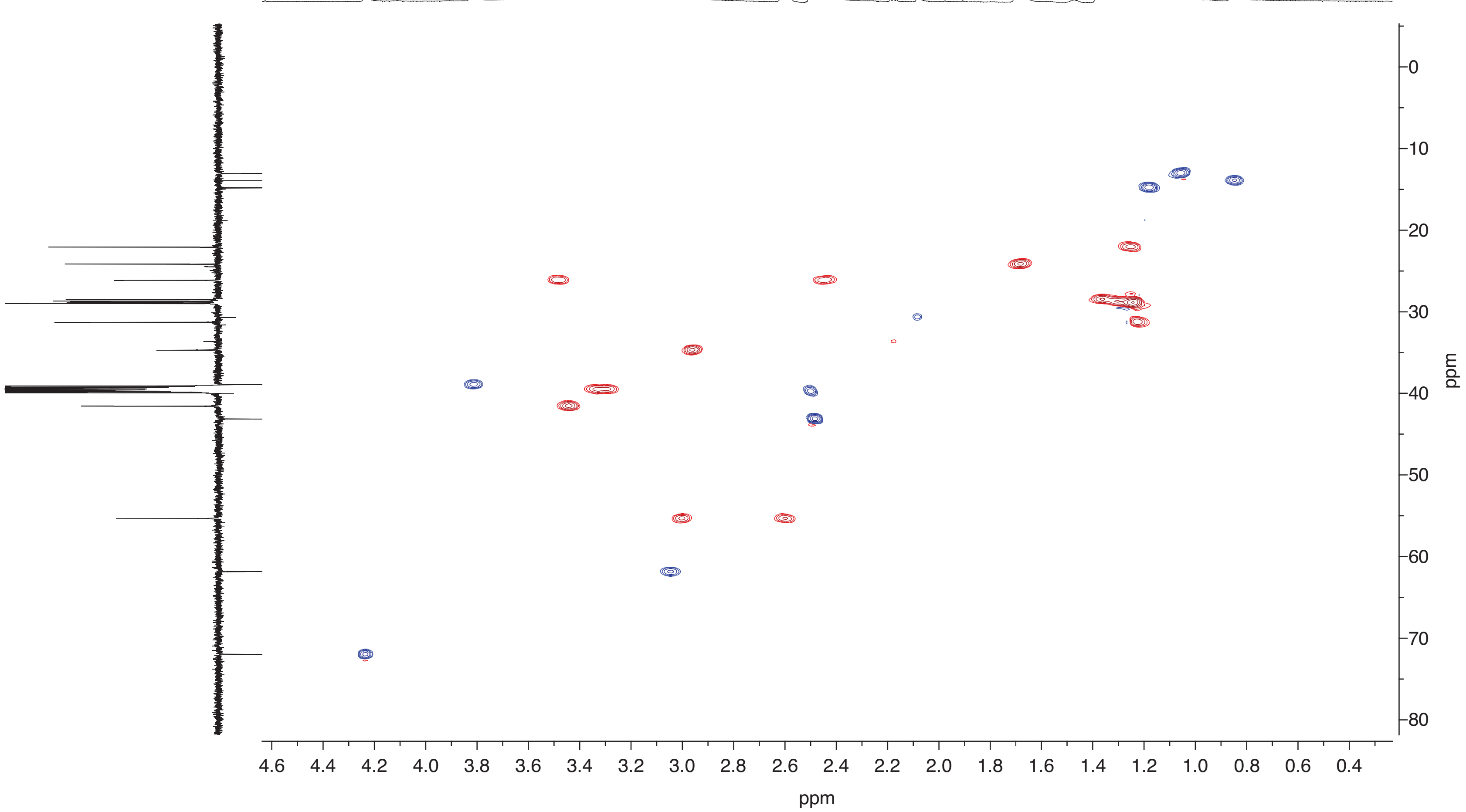

1DD-LSD tartrate (3:2)
HMBC (600/150 MHz)
DMSO-d_6

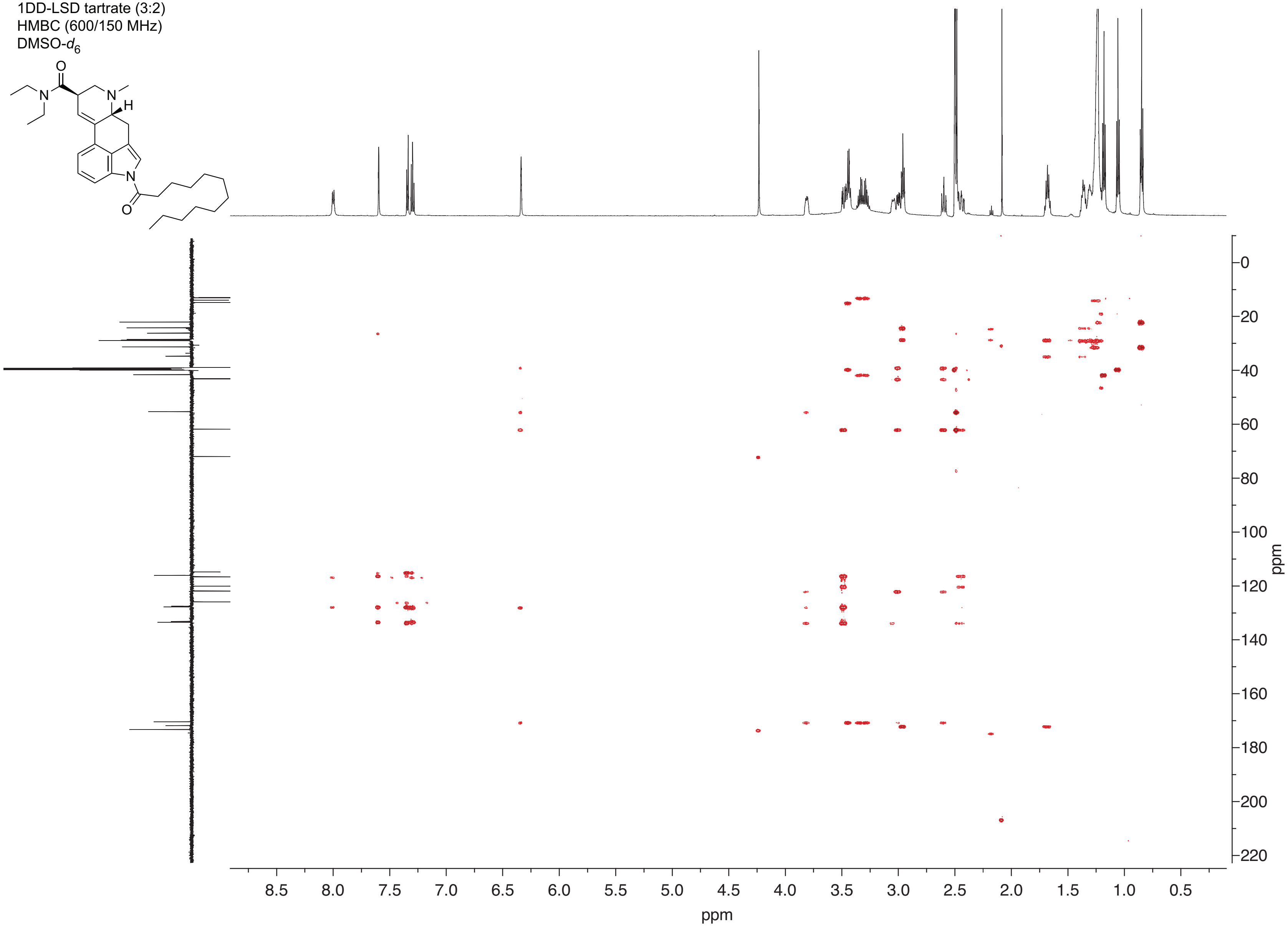

1DD-LSD tartrate (3:2)
HMBC (600/150 MHz)
DMSO-d_6

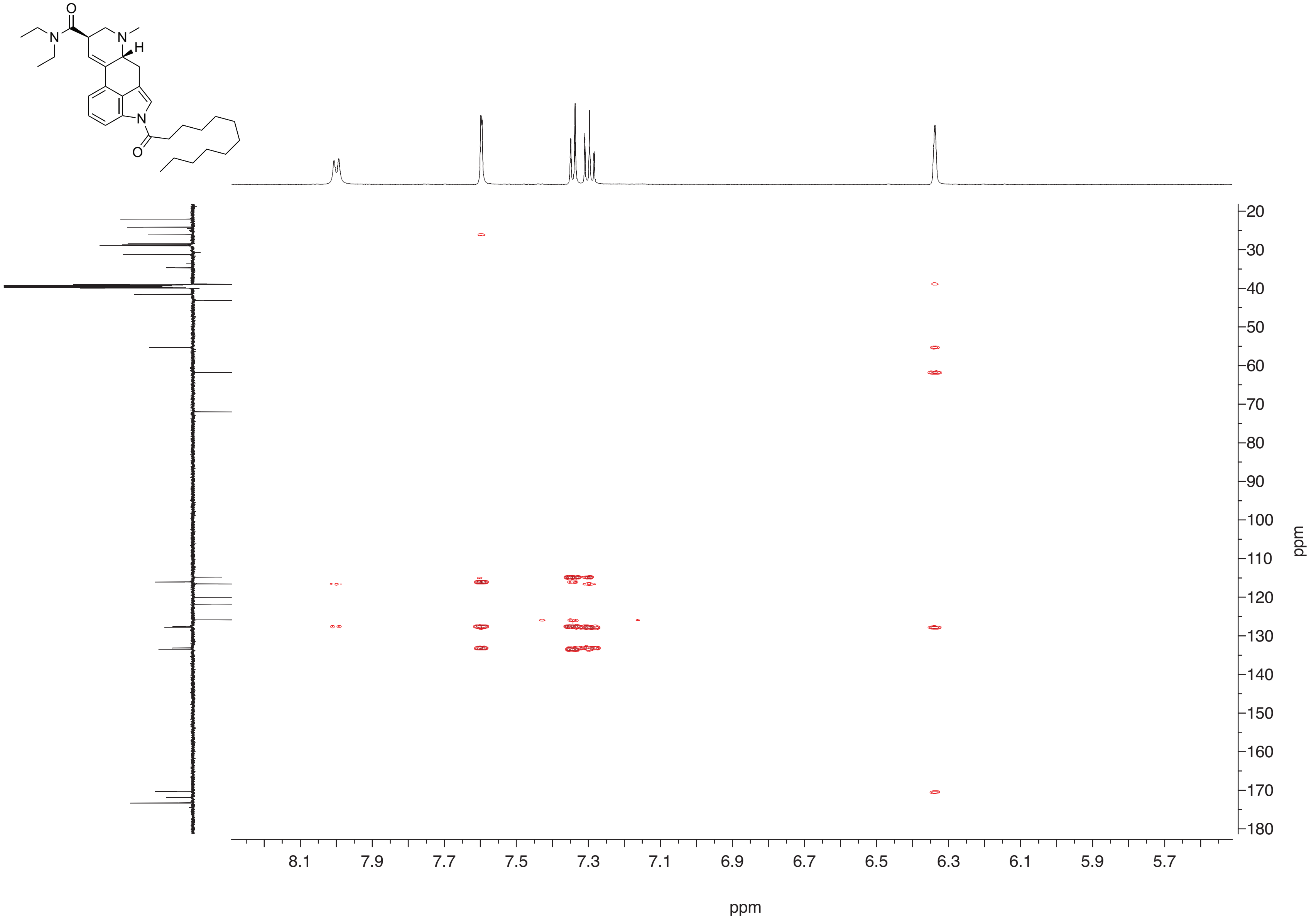

1DD-LSD tartrate (3:2)
HMBC (600/150 MHz)
DMSO-d_6

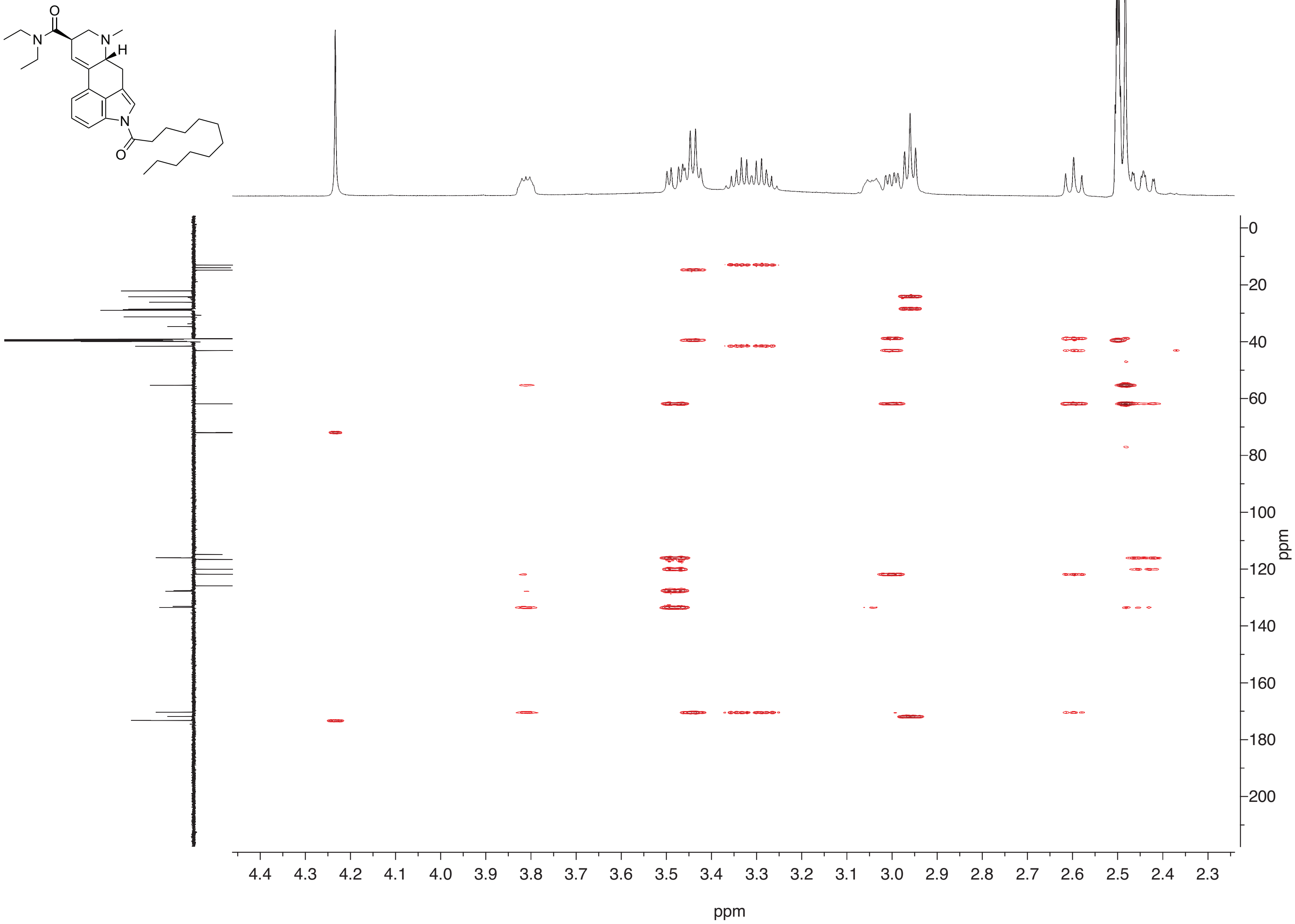

1DD-LSD tartrate (3:2)
HMBC (600/150 MHz)
DMSO-d_6

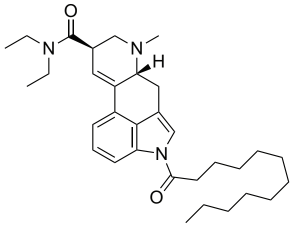

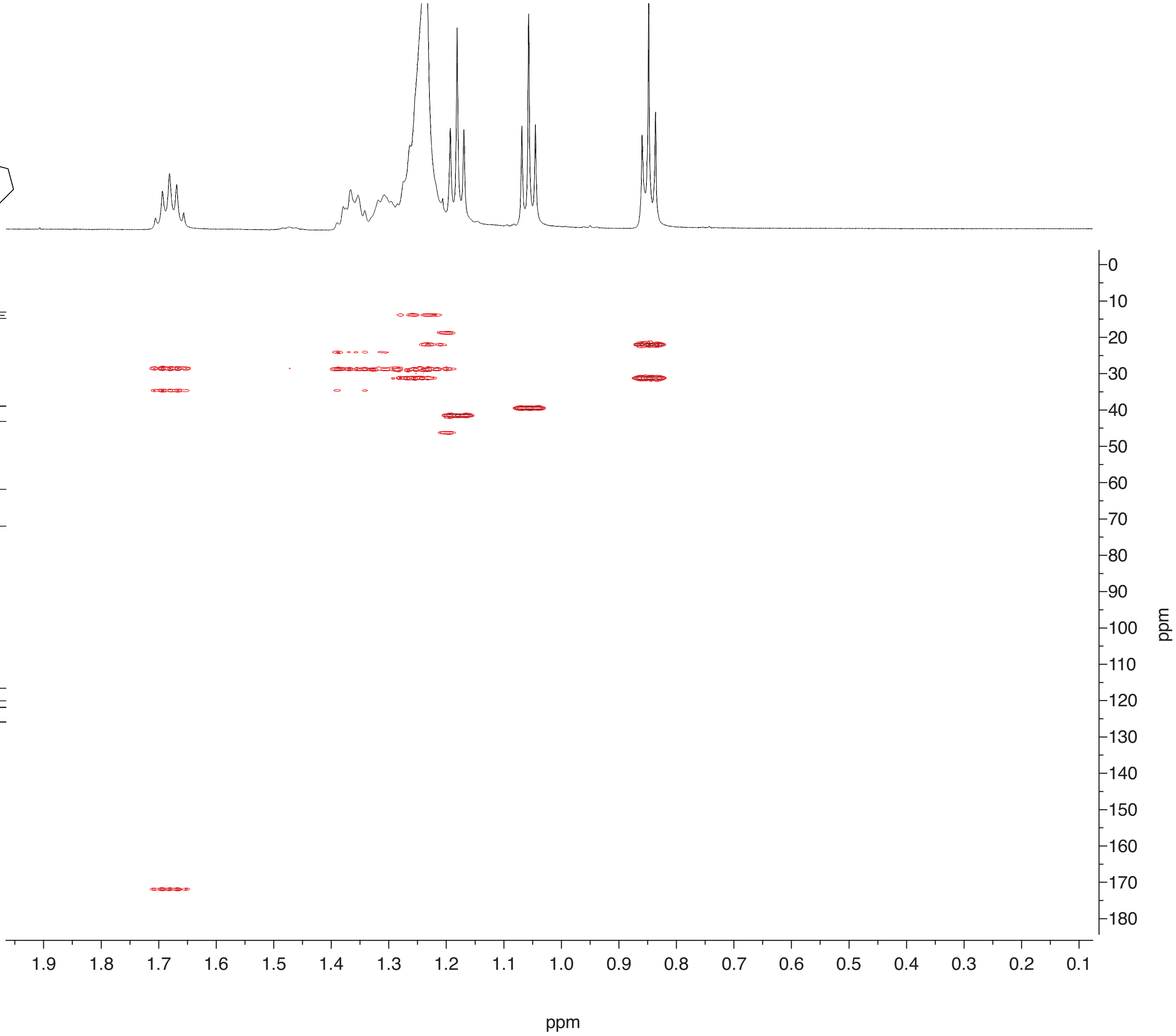

Supplement: Supplementary file 1 — Data S1. Supporting Information. [file DTA-17-101-s001.pdf]
